# Supplementary material for: Implementation of the AAMC's Holistic Review Model for Psychiatry Resident Recruitment
Source: MedEdPORTAL. 2023 Feb 7;19:11299. doi: 10.15766/mep_2374-8265.11299 (PMC9902530; doi:10.15766/mep_2374-8265.11299)
Supplement: Supplementary file 1 — Holistic Review Didactic Slides.pptxBreakout Group Exercise Worksheet.docxApplicant Criteria Identification and Prioritization.docxApplying Holistic Review to Resident Selection.docxSurvey.docx [file mep_2374-8265.11299-s001.zip › C. Applicant Criteria Identification and Prioritization.docx]

# **Applicant Criteria Identification and Prioritization**

**Purpose:** A critical part of a holistic selection process is identifying Experiences, Attributes, Competencies, and Metrics (EACMs) that are grounded in your mission and promote diversity and inclusion. Developing a shared understanding of how these criteria are prioritized facilitates recruitment, helps orient reviewers and interviewers, and informs the development of evaluation rubrics.

This activity will help you to “widen the lens” through which you assess residents by identifying and ranking the mission-driven EACMs that would add value to your program.

**Directions:** For each applicant criterion:

**Part 1**

1. Determine if each example in the following charts should be included, edited, or eliminated from your resident selection process.
2. Add any criteria that would be important to the accomplishment of your institution’s mission and program goals.

**Part 2**

1. Rank how the EACMs contribute to your decision to invite a resident for an interview.

## **Experiences**

| **➀ Criteria** | **➁ Importance of criteria to interview invitation** | | | |
| --- | --- | --- | --- | --- |
|  | **Not important** | **Somewhat important** | **Important** | **Very important** |
| Educational background |  |  |  |  |
| Community service/volunteer experience |  |  |  |  |
| Leadership roles |  |  |  |  |
| Experience with diverse populations |  |  |  |  |
| Research experience |  |  |  |  |
| Life experiences |  |  |  |  |
| Distance traveled |  |  |  |  |
| Professional associations |  |  |  |  |
| Healthcare experience |  |  |  |  |
| Experience living in a medically underserved area |  |  |  |  |
|  |  |  |  |  |
|  |  |  |  |  |
|  |  |  |  |  |
|  |  |  |  |  |
|  |  |  |  |  |
|  |  |  |  |  |

## **Attributes**

| **➀ Criteria** | **➁ Importance of criteria to interview invitation** | | | |
| --- | --- | --- | --- | --- |
|  | **Not important** | **Somewhat important** | **Important** | **Very important** |
| Professional stature |  |  |  |  |
| Cultural competence/humility |  |  |  |  |
| Integrity |  |  |  |  |
| Intellectual curiosity |  |  |  |  |
| Proficiency in language(s) spoken by patient population |  |  |  |  |
| Team-minded / team player |  |  |  |  |
| Leadership |  |  |  |  |
| Interest in the desired specialty |  |  |  |  |
|  |  |  |  |  |
|  |  |  |  |  |
|  |  |  |  |  |
|  |  |  |  |  |
|  |  |  |  |  |
|  |  |  |  |  |

## **Competencies**

| **➀ Criteria** | **➁ Importance of criteria to interview invitation** | | | |
| --- | --- | --- | --- | --- |
|  | **Not important** | **Somewhat important** | **Important** | **Very important** |
| **Interpersonal and Communication Skills (ICS)** |  |  |  |  |
| Communicate effectively with patients, families, and the public, as appropriate, across a broad range of socioeconomic and cultural backgrounds |  |  |  |  |
| Communicate effectively with physicians, other health professionals, and health related agencies |  |  |  |  |
| Work effectively as a member or leader of a health care team or other professional group |  |  |  |  |
| Act in a consultative role to other physicians and health professionals |  |  |  |  |
| Maintain comprehensive, timely, and legible medical records, if applicable. |  |  |  |  |
|  |  |  |  |  |
|  |  |  |  |  |
|  |  |  |  |  |
|  |  |  |  |  |
|  |  |  |  |  |
|  |  |  |  |  |

Examples from ACGME

## **Competencies (Continued)**

| **➀ Criteria** | **➁ Importance of criteria to interview invitation** | | | |
| --- | --- | --- | --- | --- |
|  | **Not important** | **Somewhat important** | **Important** | **Very important** |
| **Professionalism (P)** |  |  |  |  |
| Compassion, integrity, and respect for others |  |  |  |  |
| Responsiveness to patient needs that supersedes self-interest |  |  |  |  |
| Respect for patient privacy and autonomy |  |  |  |  |
| Accountability to patients, society and the profession |  |  |  |  |
| Sensitivity and responsiveness to a diverse patient population, including but not limited to diversity in gender, age, culture, race, religion, disabilities, and sexual orientation |  |  |  |  |
|  |  |  |  |  |
|  |  |  |  |  |
|  |  |  |  |  |
|  |  |  |  |  |
|  |  |  |  |  |
|  |  |  |  |  |

Examples from ACGME

## **Competencies (Continued)**

| **➀ Criteria** | **➁ Importance of criteria to interview invitation** | | | |
| --- | --- | --- | --- | --- |
|  | **Not important** | **Somewhat important** | **Important** | **Very important** |
| **Practice-Based Learning and Improvement (PBLI)** |  |  |  |  |
| Identify strengths, deficiencies, and limits in one’s knowledge and expertise (self-assessment and reflection) |  |  |  |  |
| Set learning and improvement goals |  |  |  |  |
| Identify and perform appropriate learning activities |  |  |  |  |
| Incorporate formative evaluation feedback into daily practice |  |  |  |  |
| Locate, appraise, and assimilate evidence from scientific studies related to their patients’ health problems (evidence-based medicine) |  |  |  |  |
| Use information technology to optimize learning |  |  |  |  |
| Participate in the education of patients, families, students, residents and other health professionals |  |  |  |  |
|  |  |  |  |  |
|  |  |  |  |  |
|  |  |  |  |  |
|  |  |  |  |  |
|  |  |  |  |  |
|  |  |  |  |  |

Examples from ACGM

## **Metrics**

**Note:** If these metrics are not available to you, please edit, delete, and/or add any alternatives.

| **➀ Criteria** | **➁ Importance of criteria to interview invitation** | | | |
| --- | --- | --- | --- | --- |
|  | **Not important** | **Somewhat important** | **Important** | **Very important** |
| Publications |  |  |  |  |
| Scholarly Presentations |  |  |  |  |
| USMLE Step 1 score |  |  |  |  |
| USMLE Step 1 pass on first attempt |  |  |  |  |
| USMLE Step 2CK score |  |  |  |  |
| USMLE Step 2CK pass on first attempt |  |  |  |  |
| USMLE Step 2CS |  |  |  |  |
| USMLE Step 2CS pass on first attempt |  |  |  |  |
| Alpha Omega Alpha |  |  |  |  |
| Gold Humanism Honor Society |  |  |  |  |
| Grants |  |  |  |  |
| Medical school GPA |  |  |  |  |
| Performance in core clerkships |  |  |  |  |
| Clerkship performance in desired specialty |  |  |  |  |
| Honors in curriculum |  |  |  |  |
| MSPE |  |  |  |  |
| Letters of recommendation |  |  |  |  |
|  |  |  |  |  |
|  |  |  |  |  |
|  |  |  |  |  |
|  |  |  |  |  |
|  |  |  |  |  |
|  |  |  |  |  |
