# Supplementary material for: Implementation of the AAMC's Holistic Review Model for Psychiatry Resident Recruitment
Source: MedEdPORTAL. 2023 Feb 7;19:11299. doi: 10.15766/mep_2374-8265.11299 (PMC9902530; doi:10.15766/mep_2374-8265.11299)
Supplement: Supplementary file 1 — Holistic Review Didactic Slides.pptxBreakout Group Exercise Worksheet.docxApplicant Criteria Identification and Prioritization.docxApplying Holistic Review to Resident Selection.docxSurvey.docx [file mep_2374-8265.11299-s001.zip › D. Applying Holistic Review to Resident Selection.docx]

# **Applying Holistic Review to Resident Selection**

**Purpose:** Developing shared definitions of the criteria you identified in Activity 1 helps orient reviewers and interviewers and informs the development of evaluation rubrics; it can also help mitigate the influence of unconscious bias. This activity will help you to define your high-priority criteria and assess if your recruitment materials and selection processes reflect your priorities.

**Directions:**

1. Review your rankings from Activity 1 and select two “very important” criteria for each of the four domains in the EACM model. Clearly define each of those criteria.
2. Look at your current recruitment materials and selection filters to determine if these will reveal the priority criteria that you have identified.
3. Determine what you could add or change to assist you in finding the EACMs you are looking for.

## **Part 1: Resident Selection Criteria**

| **EXPERIENCES** | **1.** *Criterion:* |
| --- | --- |
|  | **Definition:** *How do you define it?* |
|  | **Assess:** *What evidence will satisfy this requirement? Do my current recruitment and selection materials allow me to assess this criterion? What, if any, changes are needed?* |
|  |  |
|  | **2.** *Criterion:* |
|  | **Definition:** *How do you define it?* |
|  | **Assess:** *What evidence will satisfy this requirement? Do my current recruitment and selection materials allow me to assess this criterion? What, if any, changes are needed?* |

**Notes:**

## **Resident Selection Criteria (continued)**

| **ATTRIBUTES** | **1.** *Criterion:* |
| --- | --- |
|  | **Definition:** *How do you define it?* |
|  | **Assess:** *What evidence will satisfy this requirement? Do my current recruitment and selection materials allow me to assess this criterion? What, if any, changes are needed?* |
|  |  |
|  | **2.** *Criterion:* |
|  | **Definition:** *How do you define it?* |
|  | **Assess:** *What evidence will satisfy this requirement? Do my current recruitment and selection materials allow me to assess this criterion? What, if any, changes are needed?* |

**Notes:**

## **Resident Selection Criteria (continued)**

| **COMPETENCIES** | **1.** *Criterion:* |
| --- | --- |
|  | **Definition:** *How do you define it?* |
|  | **Assess:** *What evidence will satisfy this requirement? Do my current recruitment and selection materials allow me to assess this criterion? What, if any, changes are needed?* |
|  |  |
|  | **2.** *Criterion:* |
|  | **Definition:** *How do you define it?* |
|  | **Assess:** *What evidence will satisfy this requirement? Do my current recruitment and selection materials allow me to assess this criterion? What, if any, changes are needed?* |

**Notes:**

## **Resident Selection Criteria (continued)**

| **ACADEMIC METRICS** | **1.** *Criterion:* |
| --- | --- |
|  | **Definition:** *How do you define it?* |
|  | **Assess:** *What evidence will satisfy this requirement? Do my current recruitment and selection materials allow me to assess this criterion? What, if any, changes are needed?* |
|  |  |
|  | **2.** *Criterion:* |
|  | **Definition:** *How do you define it?* |
|  | **Assess:** *What evidence will satisfy this requirement? Do my current recruitment and selection materials allow me to assess this criterion? What, if any, changes are needed?* |

**Notes:**
