# Supplementary material for: Implementation of the AAMC's Holistic Review Model for Psychiatry Resident Recruitment
Source: MedEdPORTAL. 2023 Feb 7;19:11299. doi: 10.15766/mep_2374-8265.11299 (PMC9902530; doi:10.15766/mep_2374-8265.11299)
Supplement: Supplementary file 1 — Holistic Review Didactic Slides.pptxBreakout Group Exercise Worksheet.docxApplicant Criteria Identification and Prioritization.docxApplying Holistic Review to Resident Selection.docxSurvey.docx [file mep_2374-8265.11299-s001.zip › E. Survey.docx]

Appendix E: Survey

These questions were asked using the virtual platform survey tools and developed by the authors of the workshop:

| What stage are you in implementing holistic review? | |
| --- | --- |
|  | What is holistic review? |
|  | Have not started |
|  | Reviewed but not started |
|  | Used for < 1 year |
|  | Used 1+ years (teaching holistic review to others; Milestone Level 5) |
|  | |
| What are challenges to resident selection at your program related to implementing a holistic review process? | |
|  | |
|  | |
| What are challenges for ranking these attributes/experiences? | |
|  | |
|  | |
| How likely are you to implement holistic application to your next application cycle? | |
|  | Extremely Unlikely |
|  | Unlikely |
|  | Neutral |
|  | Likely |
|  | Extremely Likely |

These questions were asked as part the conference survey:

| Y/N |  |
| --- | --- |
|  | Were you able to take away at least one actionable item? |
|  | Was the workshop session interactive? |
|  | Did the workshop session meet your expectations? |
