# Supplementary material for: Implementation of the AAMC's Holistic Review Model for Psychiatry Resident Recruitment
Source: MedEdPORTAL. 2023 Feb 7;19:11299. doi: 10.15766/mep_2374-8265.11299 (PMC9902530; doi:10.15766/mep_2374-8265.11299)
Supplement: Supplementary file 1 — Holistic Review Didactic Slides.pptxBreakout Group Exercise Worksheet.docxApplicant Criteria Identification and Prioritization.docxApplying Holistic Review to Resident Selection.docxSurvey.docx [file mep_2374-8265.11299-s001.zip › A. Holistic Review Didactic Slides.pptx]

## Slide 1
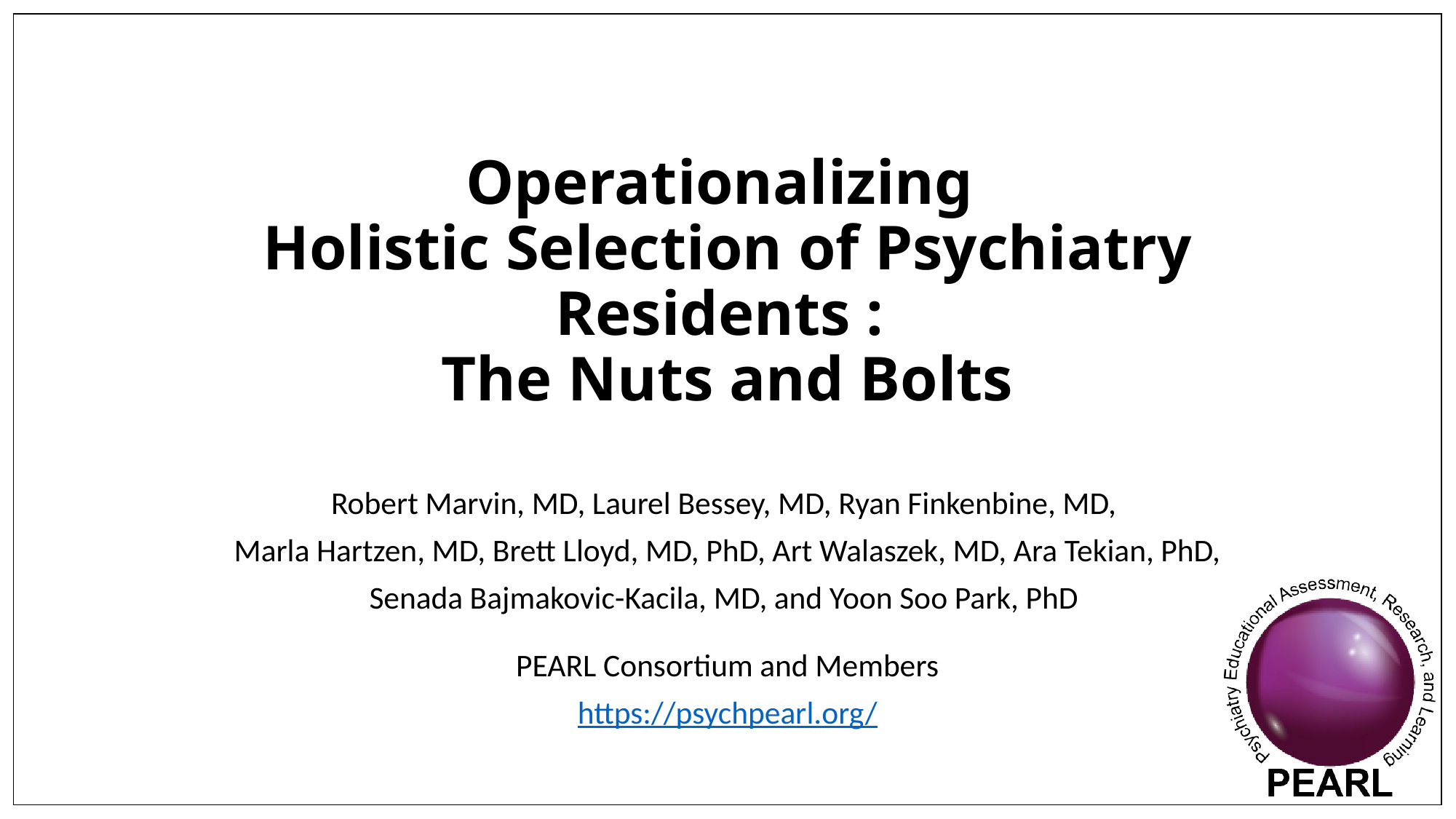

# Operationalizing Holistic Selection of Psychiatry Residents : The Nuts and Bolts
Robert Marvin, MD, Laurel Bessey, MD, Ryan Finkenbine, MD,
Marla Hartzen, MD, Brett Lloyd, MD, PhD, Art Walaszek, MD, Ara Tekian, PhD,
Senada Bajmakovic-Kacila, MD, and Yoon Soo Park, PhD
PEARL Consortium and Members
https://psychpearl.org/

## Slide 2
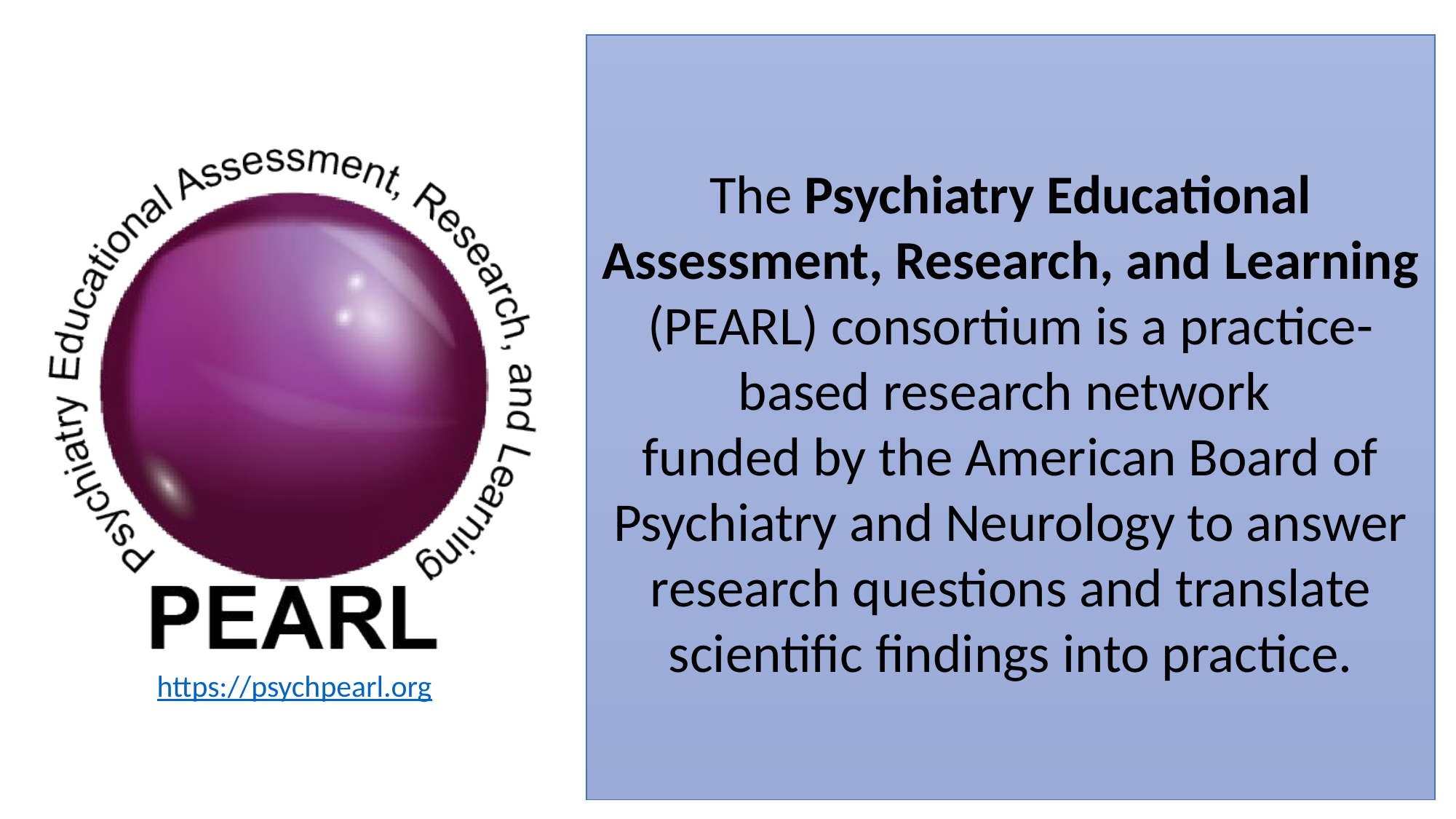

The Psychiatry Educational Assessment, Research, and Learning (PEARL) consortium is a practice-based research network
funded by the American Board of Psychiatry and Neurology to answer research questions and translate scientific findings into practice.
https://psychpearl.org

## Slide 3
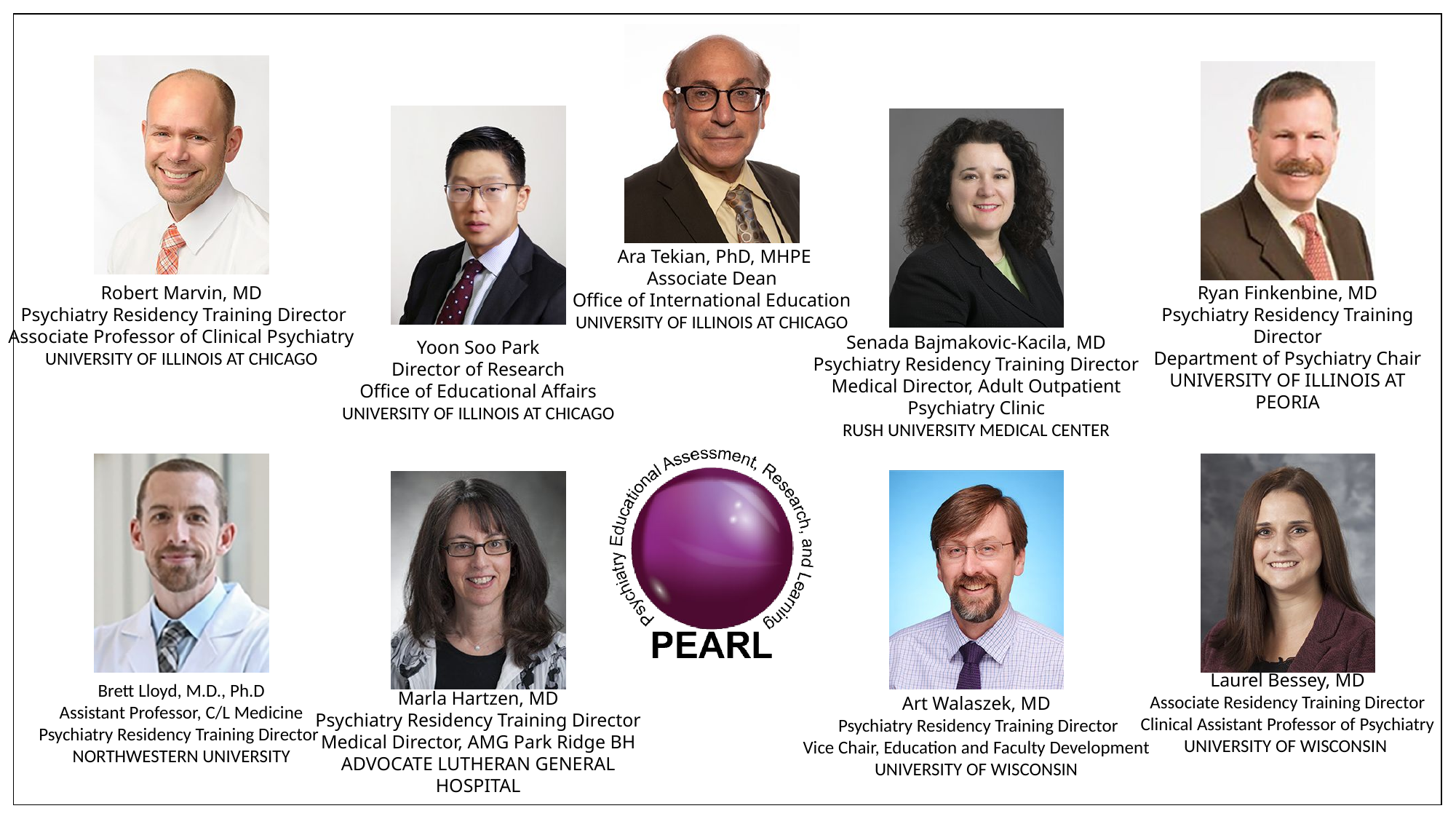

Ara Tekian, PhD, MHPE
Associate Dean
Office of International Education
UNIVERSITY OF ILLINOIS AT CHICAGO
Robert Marvin, MD
 Psychiatry Residency Training Director
Associate Professor of Clinical Psychiatry
UNIVERSITY OF ILLINOIS AT CHICAGO
Ryan Finkenbine, MD
Psychiatry Residency Training Director
Department of Psychiatry Chair
UNIVERSITY OF ILLINOIS AT PEORIA
Senada Bajmakovic-Kacila, MD
Psychiatry Residency Training Director
Medical Director, Adult Outpatient Psychiatry Clinic
RUSH UNIVERSITY MEDICAL CENTER
Yoon Soo Park
Director of Research
Office of Educational Affairs
UNIVERSITY OF ILLINOIS AT CHICAGO
Laurel Bessey, MD
Associate Residency Training Director
Clinical Assistant Professor of Psychiatry
UNIVERSITY OF WISCONSIN
Brett Lloyd, M.D., Ph.D
Assistant Professor, C/L Medicine
Psychiatry Residency Training Director
NORTHWESTERN UNIVERSITY
Marla Hartzen, MD
Psychiatry Residency Training Director
Medical Director, AMG Park Ridge BH
ADVOCATE LUTHERAN GENERAL HOSPITAL
Art Walaszek, MD
 Psychiatry Residency Training Director
Vice Chair, Education and Faculty Development
 UNIVERSITY OF WISCONSIN

## Slide 4
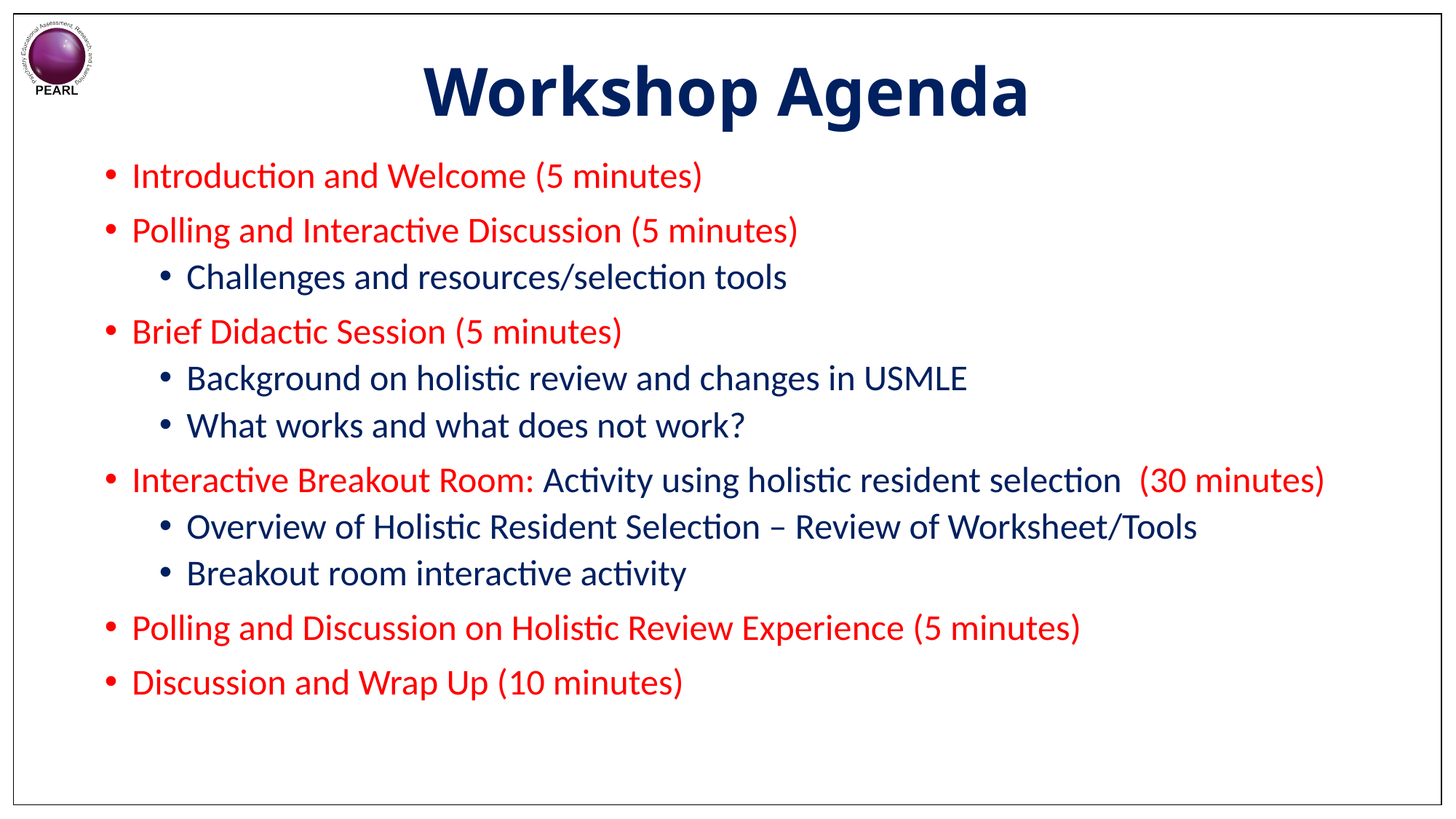

# Workshop Agenda
Introduction and Welcome (5 minutes)
Polling and Interactive Discussion (5 minutes)
Challenges and resources/selection tools
Brief Didactic Session (5 minutes)
Background on holistic review and changes in USMLE
What works and what does not work?
Interactive Breakout Room: Activity using holistic resident selection (30 minutes)
Overview of Holistic Resident Selection – Review of Worksheet/Tools
Breakout room interactive activity
Polling and Discussion on Holistic Review Experience (5 minutes)
Discussion and Wrap Up (10 minutes)

## Slide 5
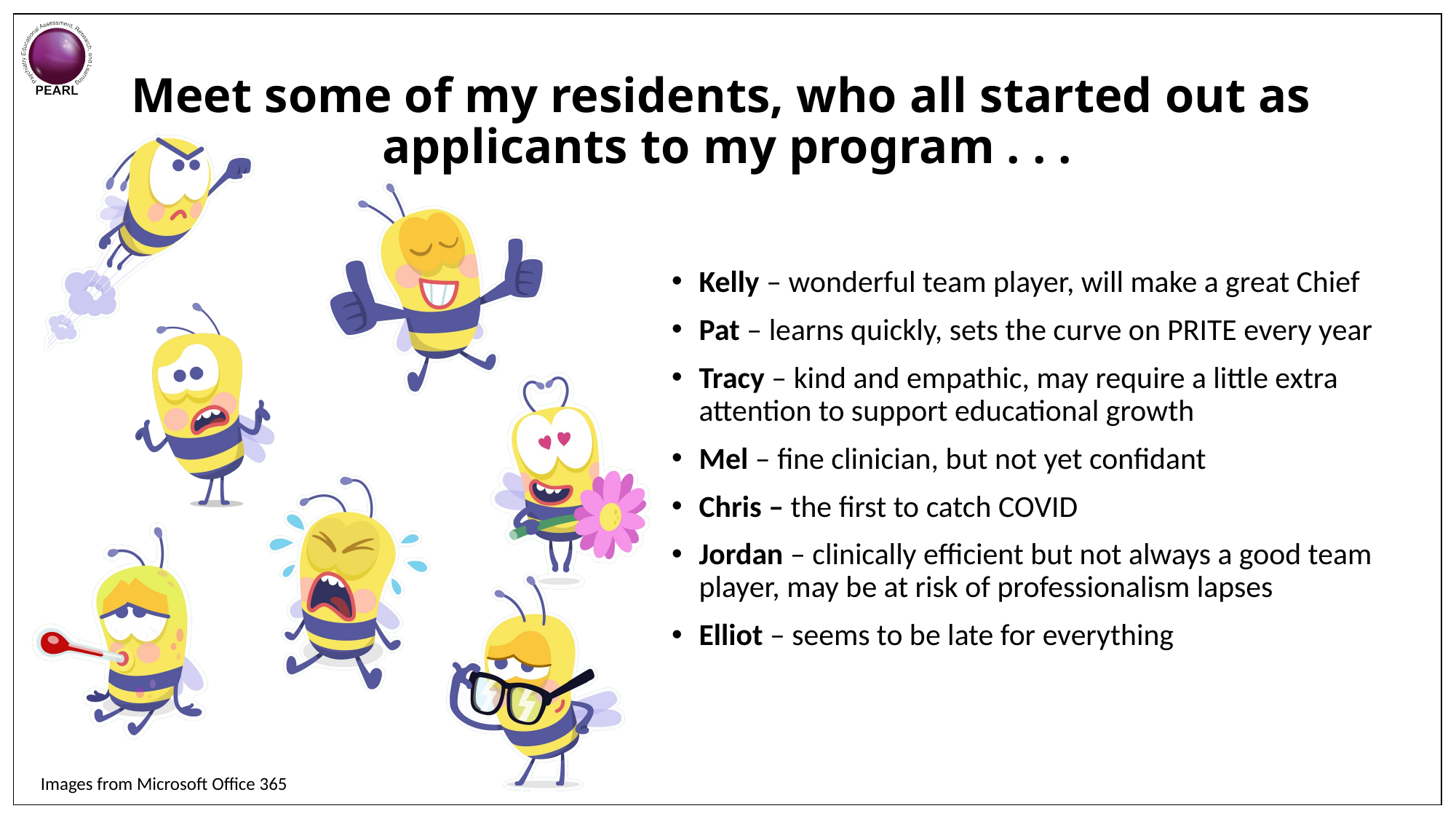

# Meet some of my residents, who all started out as applicants to my program . . .
Kelly – wonderful team player, will make a great Chief
Pat – learns quickly, sets the curve on PRITE every year
Tracy – kind and empathic, may require a little extra attention to support educational growth
Mel – fine clinician, but not yet confidant
Chris – the first to catch COVID
Jordan – clinically efficient but not always a good team player, may be at risk of professionalism lapses
Elliot – seems to be late for everything
Images from Microsoft Office 365

## Slide 6
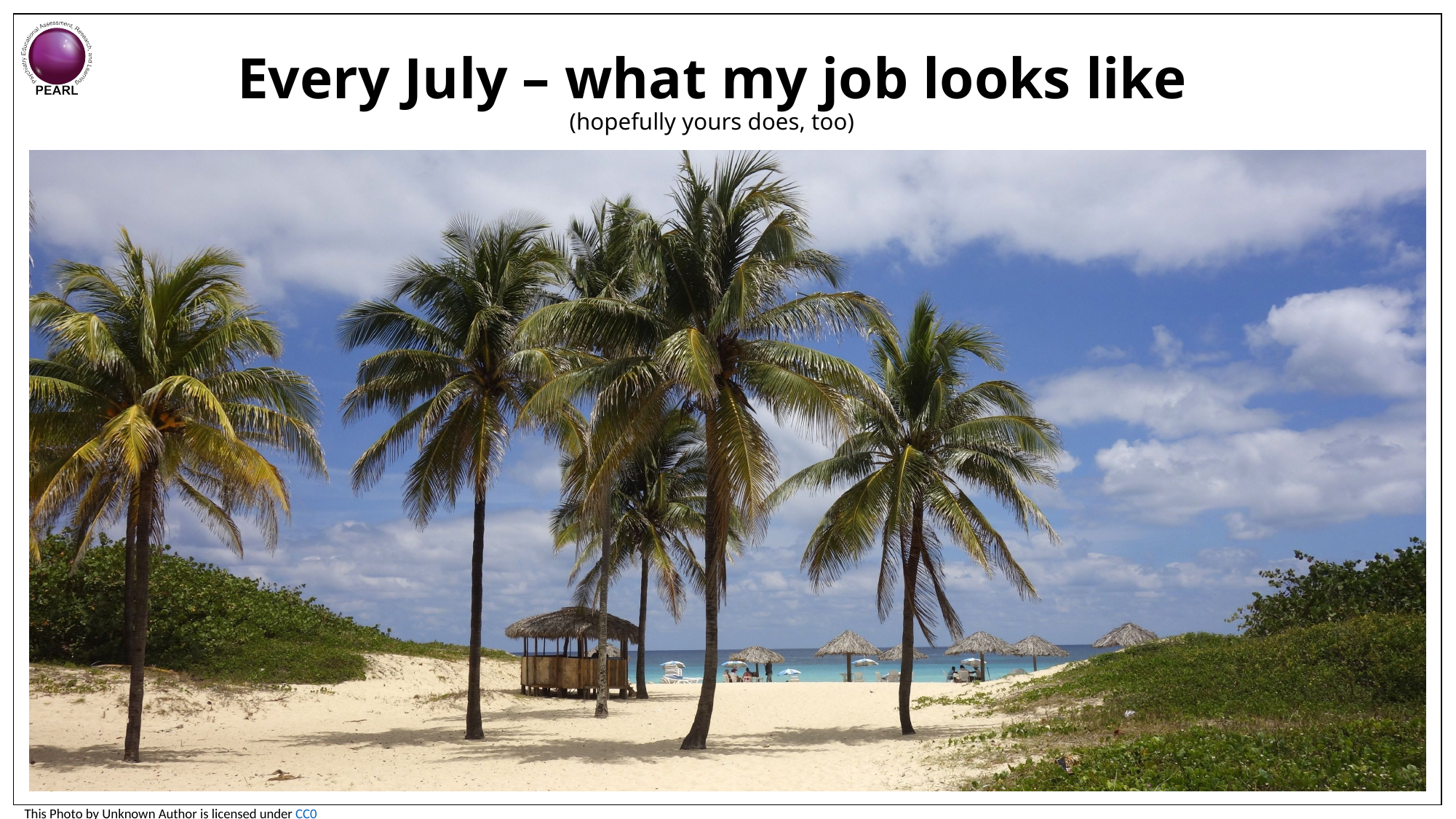

# Every July – what my job looks like (hopefully yours does, too)
This Photo by Unknown Author is licensed under CC0

## Slide 7
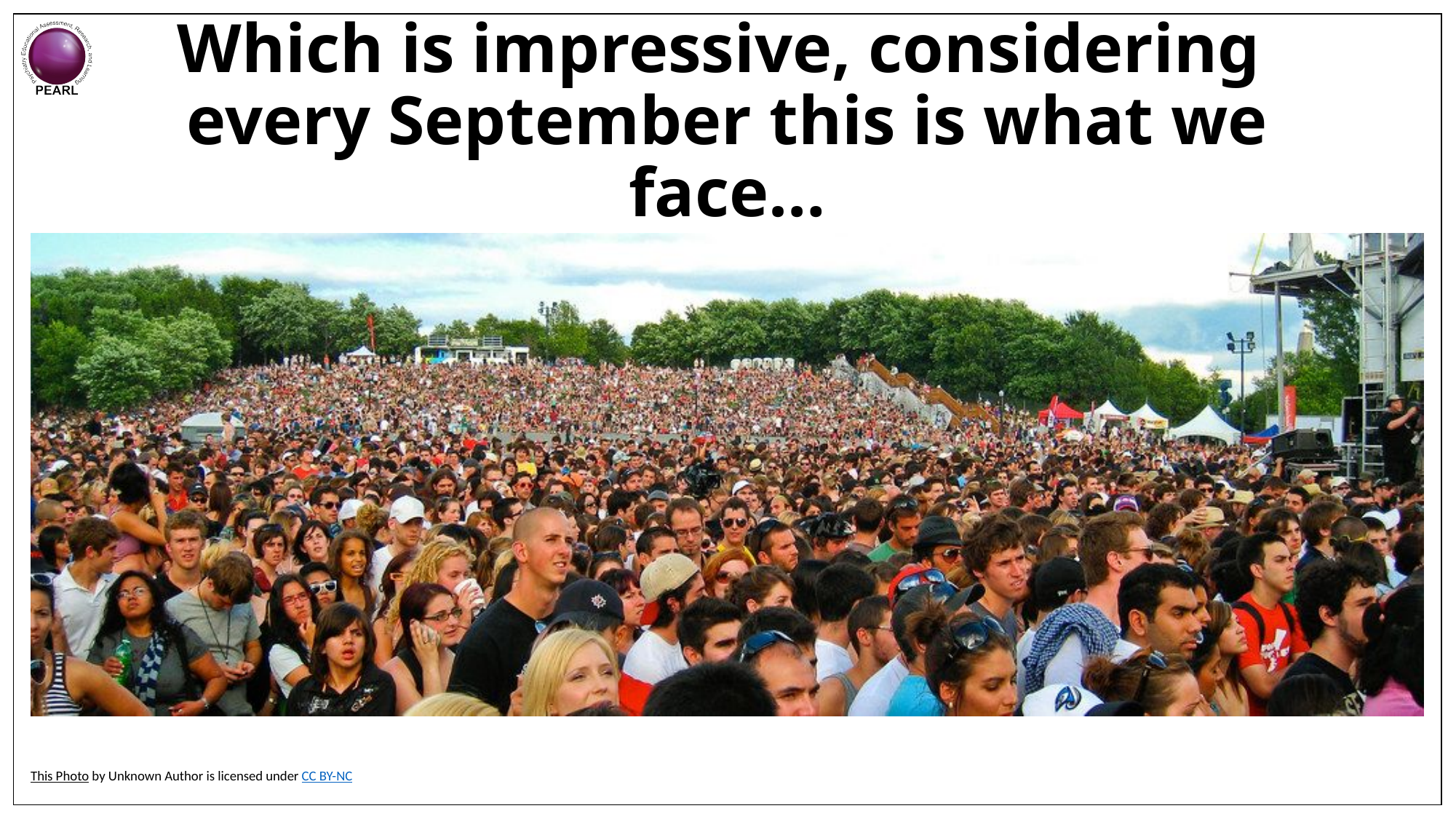

# Which is impressive, considering every September this is what we face…
This Photo by Unknown Author is licensed under CC BY-NC

## Slide 8
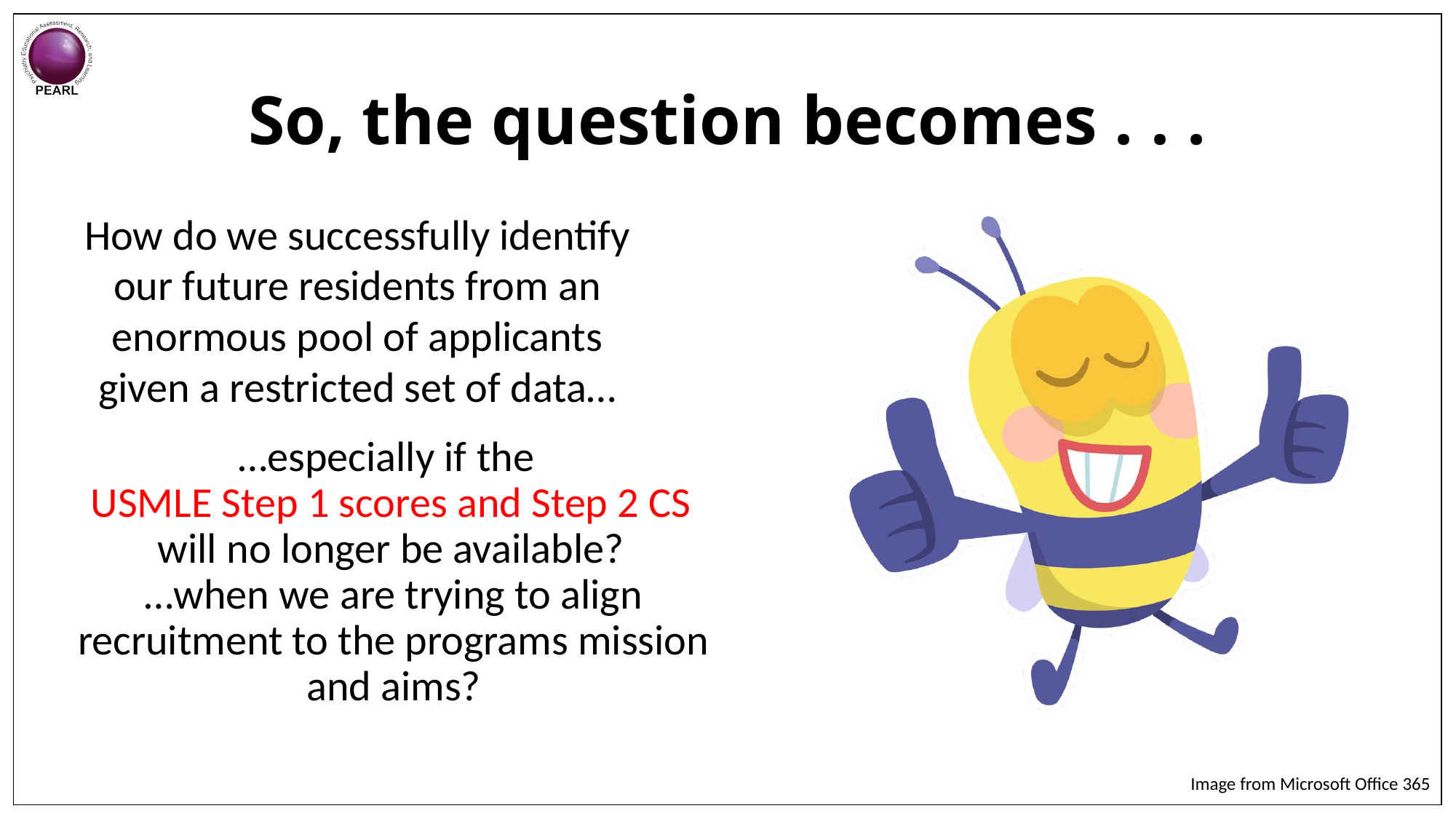

# So, the question becomes . . .
How do we successfully identify our future residents from an enormous pool of applicants given a restricted set of data…
…especially if the
USMLE Step 1 scores and Step 2 CS will no longer be available?
…when we are trying to align recruitment to the programs mission and aims?
Image from Microsoft Office 365

## Slide 9
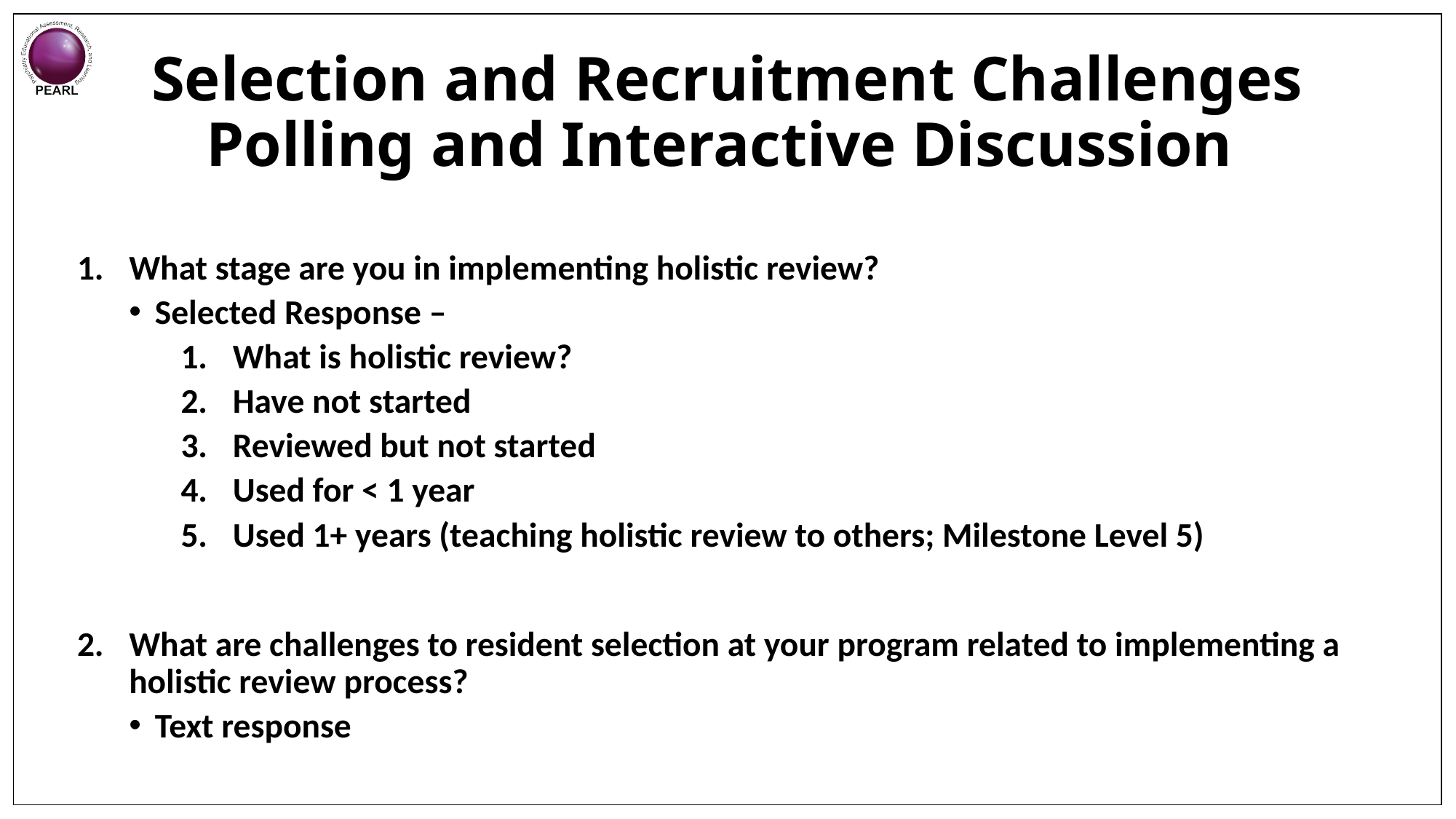

# Selection and Recruitment Challenges Polling and Interactive Discussion
What stage are you in implementing holistic review?
Selected Response –
What is holistic review?
Have not started
Reviewed but not started
Used for < 1 year
Used 1+ years (teaching holistic review to others; Milestone Level 5)
What are challenges to resident selection at your program related to implementing a holistic review process?
Text response

## Slide 10
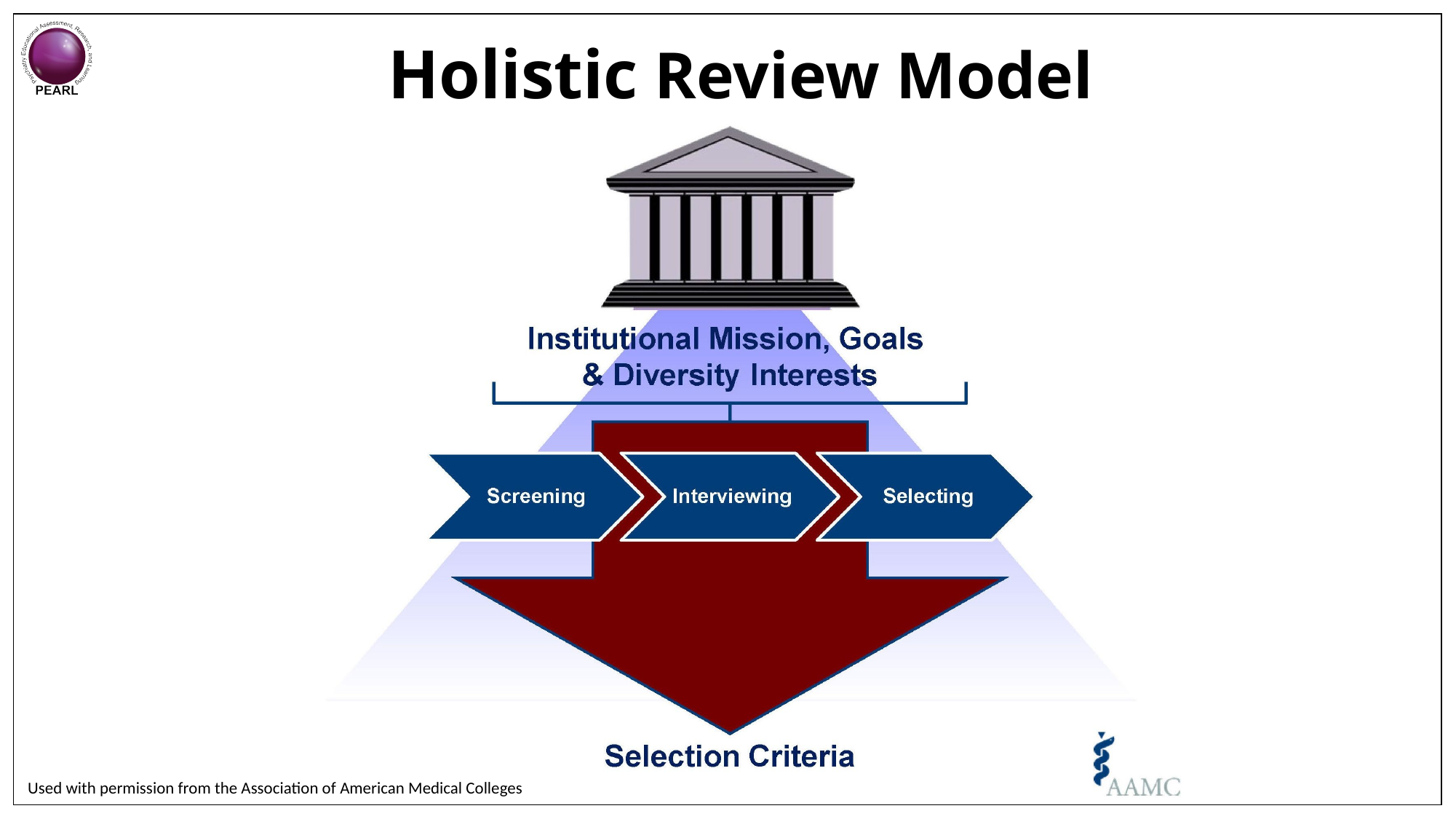

# Holistic Review Model
Used with permission from the Association of American Medical Colleges

## Slide 11
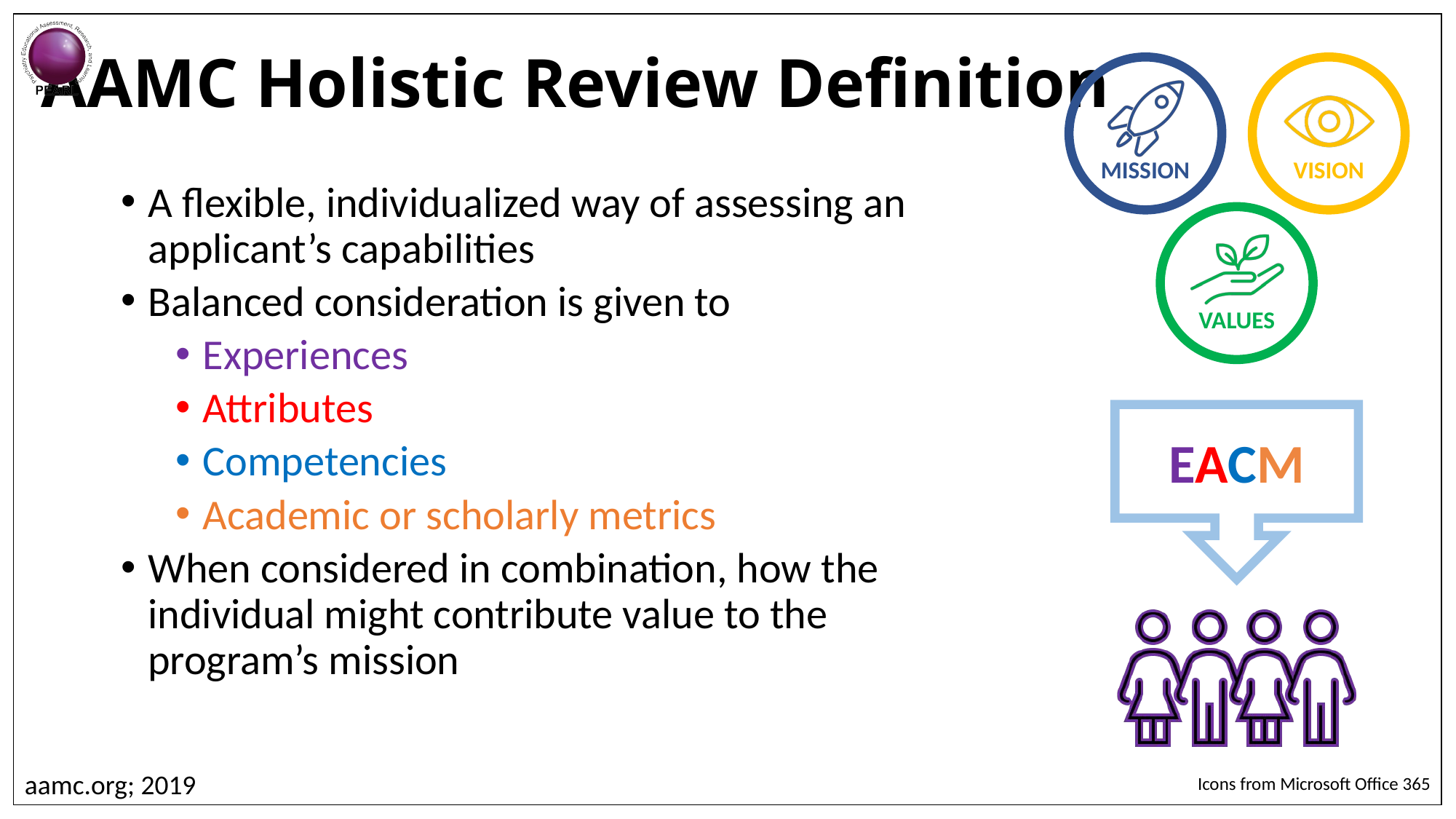

# AAMC Holistic Review Definition
MISSION
VISION
A flexible, individualized way of assessing an applicant’s capabilities
Balanced consideration is given to
Experiences
Attributes
Competencies
Academic or scholarly metrics
When considered in combination, how the individual might contribute value to the program’s mission
VALUES
EACM
aamc.org; 2019
Icons from Microsoft Office 365

## Slide 12
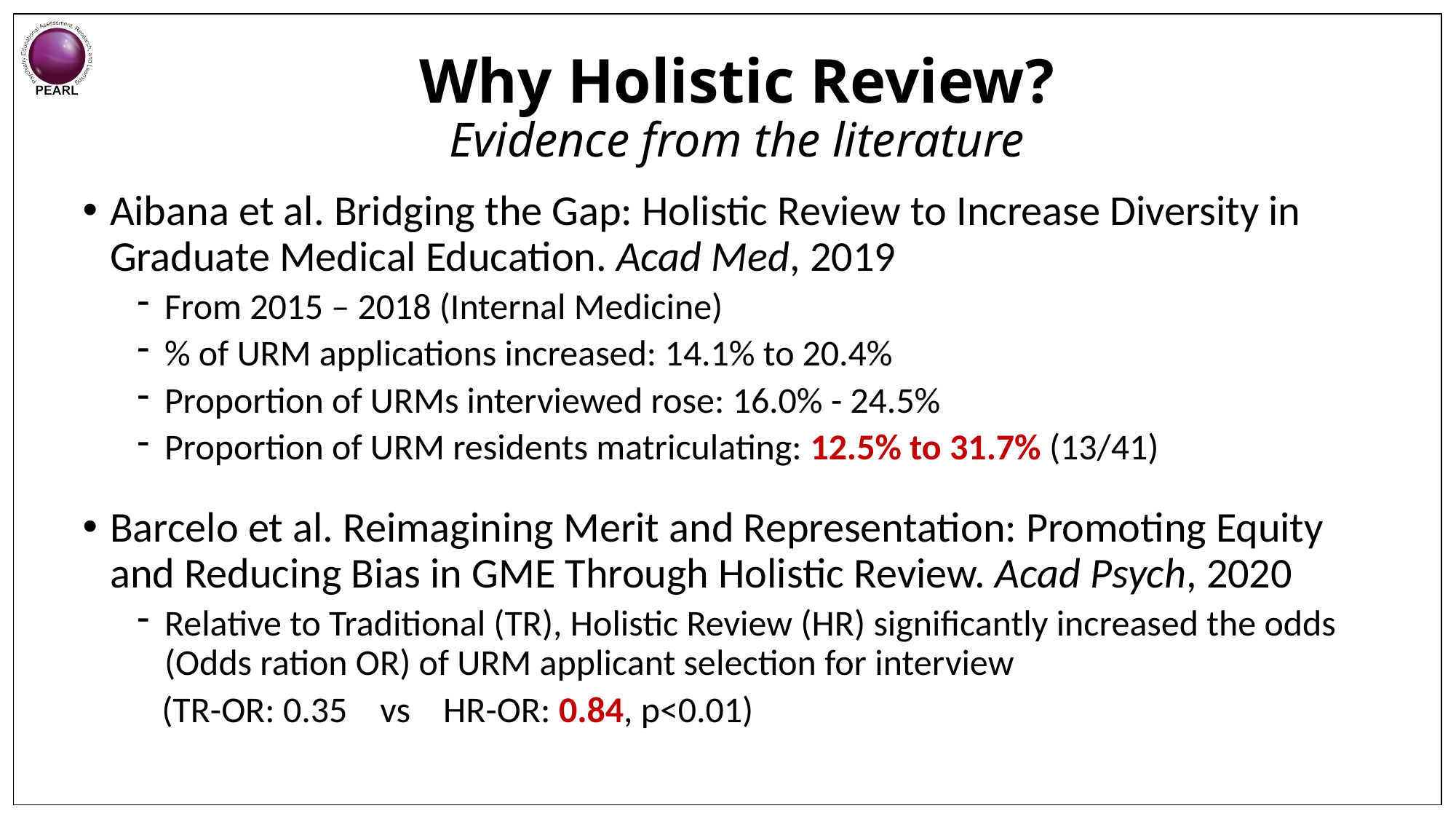

# Why Holistic Review? Evidence from the literature
Aibana et al. Bridging the Gap: Holistic Review to Increase Diversity in Graduate Medical Education. Acad Med, 2019
From 2015 – 2018 (Internal Medicine)
% of URM applications increased: 14.1% to 20.4%
Proportion of URMs interviewed rose: 16.0% - 24.5%
Proportion of URM residents matriculating: 12.5% to 31.7% (13/41)
Barcelo et al. Reimagining Merit and Representation: Promoting Equity and Reducing Bias in GME Through Holistic Review. Acad Psych, 2020
Relative to Traditional (TR), Holistic Review (HR) significantly increased the odds (Odds ration OR) of URM applicant selection for interview
 (TR-OR: 0.35 vs HR-OR: 0.84, p<0.01)

## Slide 13
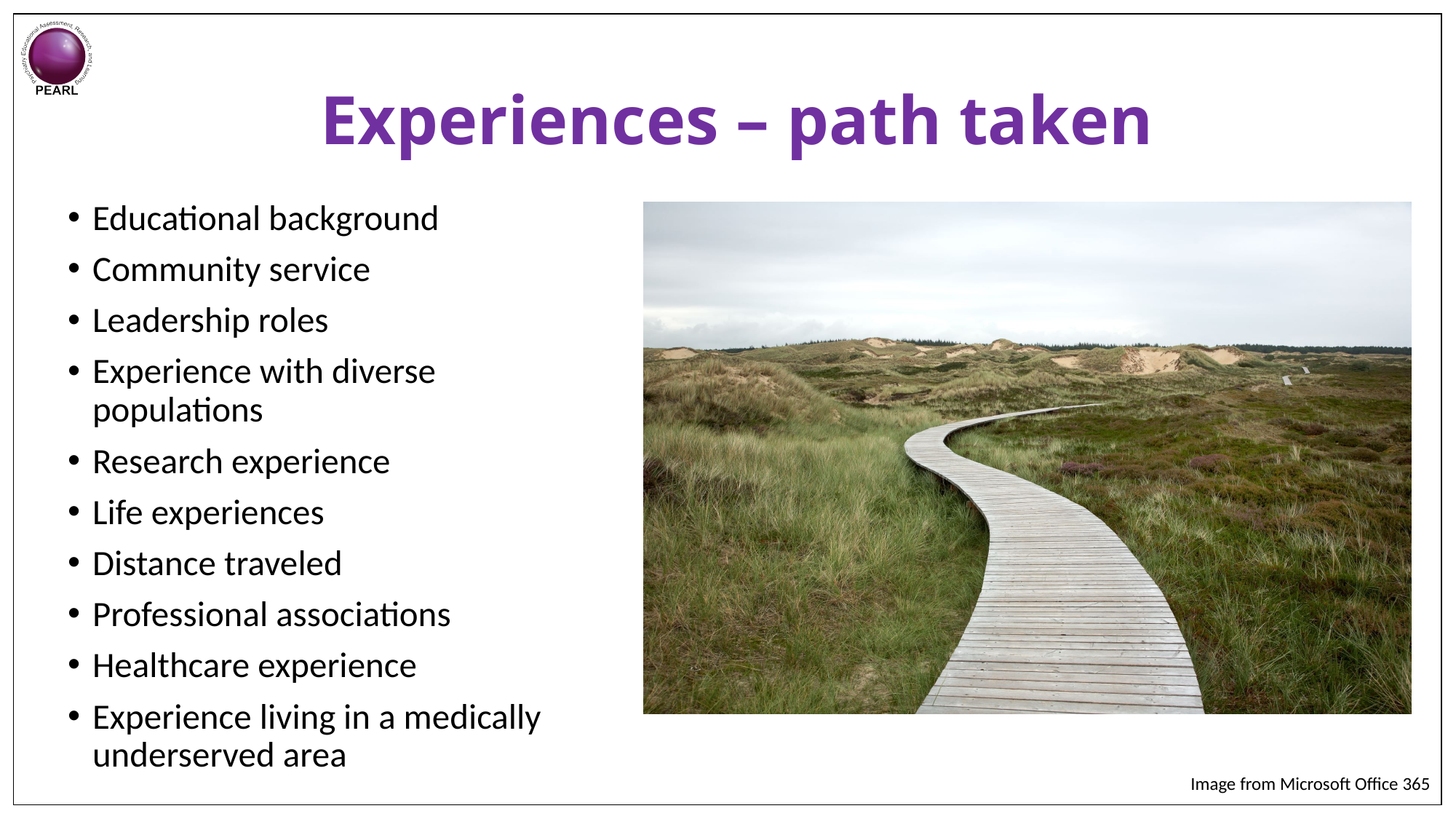

# Experiences – path taken
Educational background
Community service
Leadership roles
Experience with diverse populations
Research experience
Life experiences
Distance traveled
Professional associations
Healthcare experience
Experience living in a medically underserved area
Image from Microsoft Office 365

## Slide 14
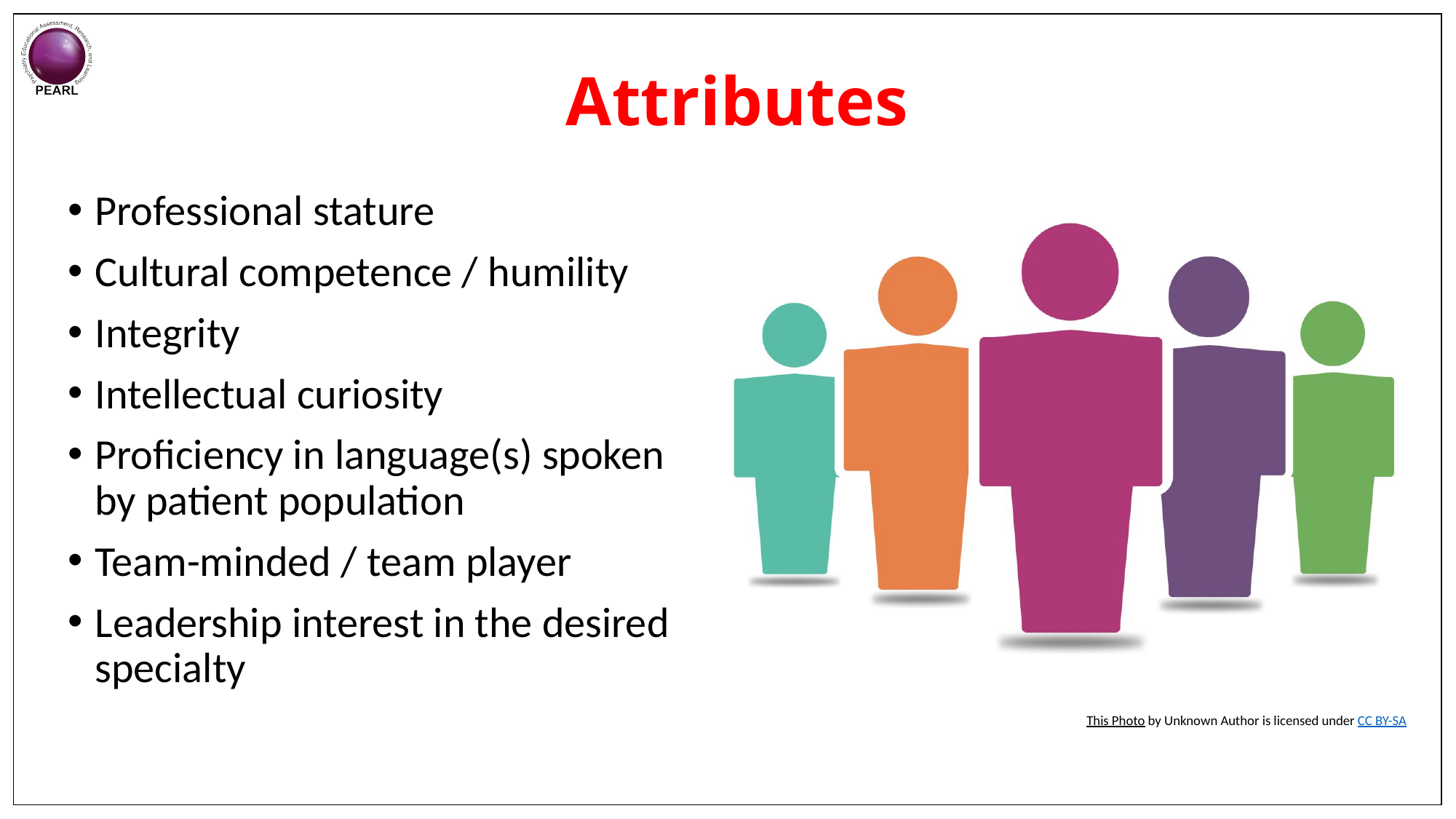

# Attributes
Professional stature
Cultural competence / humility
Integrity
Intellectual curiosity
Proficiency in language(s) spoken by patient population
Team-minded / team player
Leadership interest in the desired specialty
This Photo by Unknown Author is licensed under CC BY-SA

## Slide 15
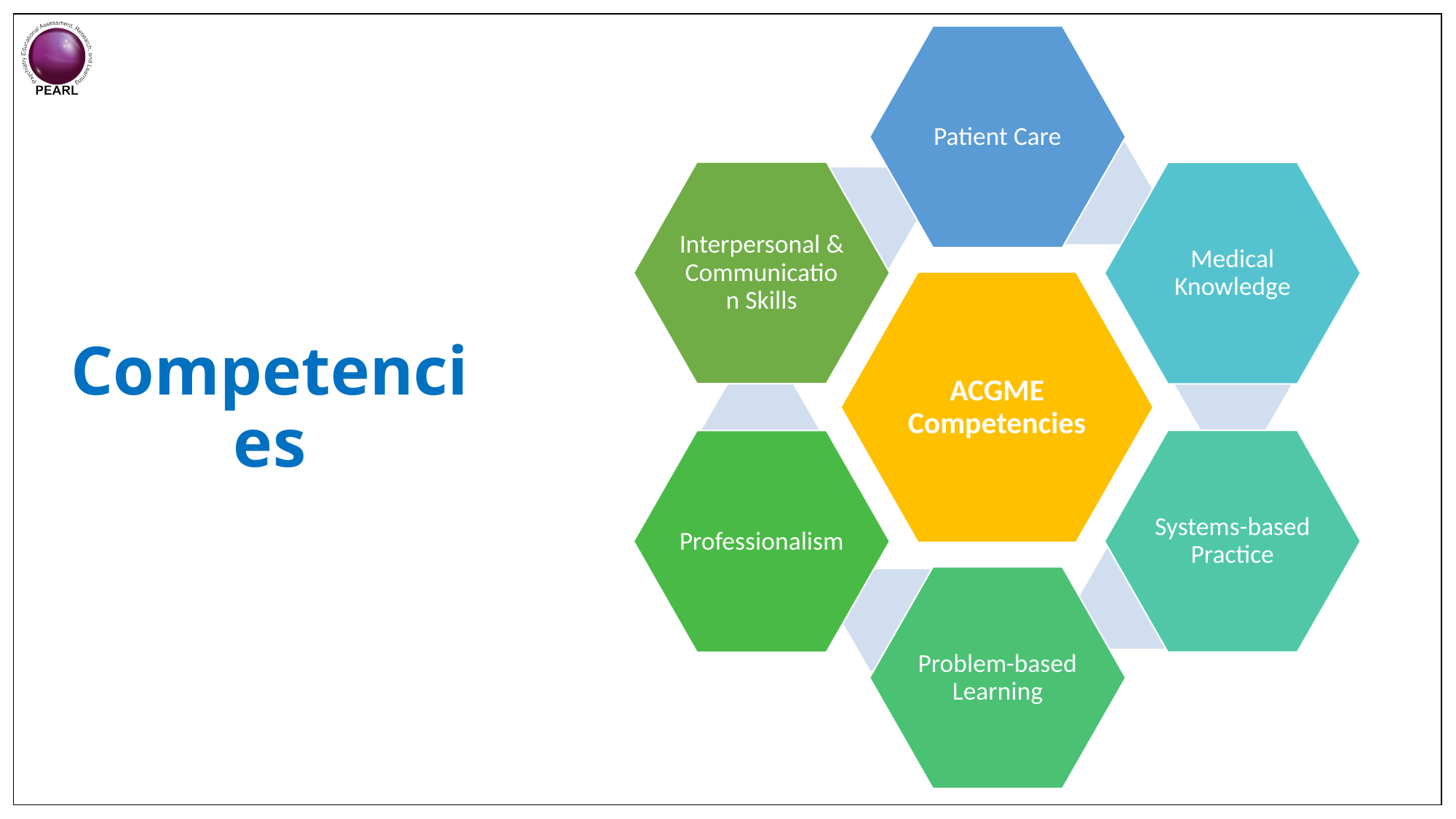

# Competencies

## Slide 16
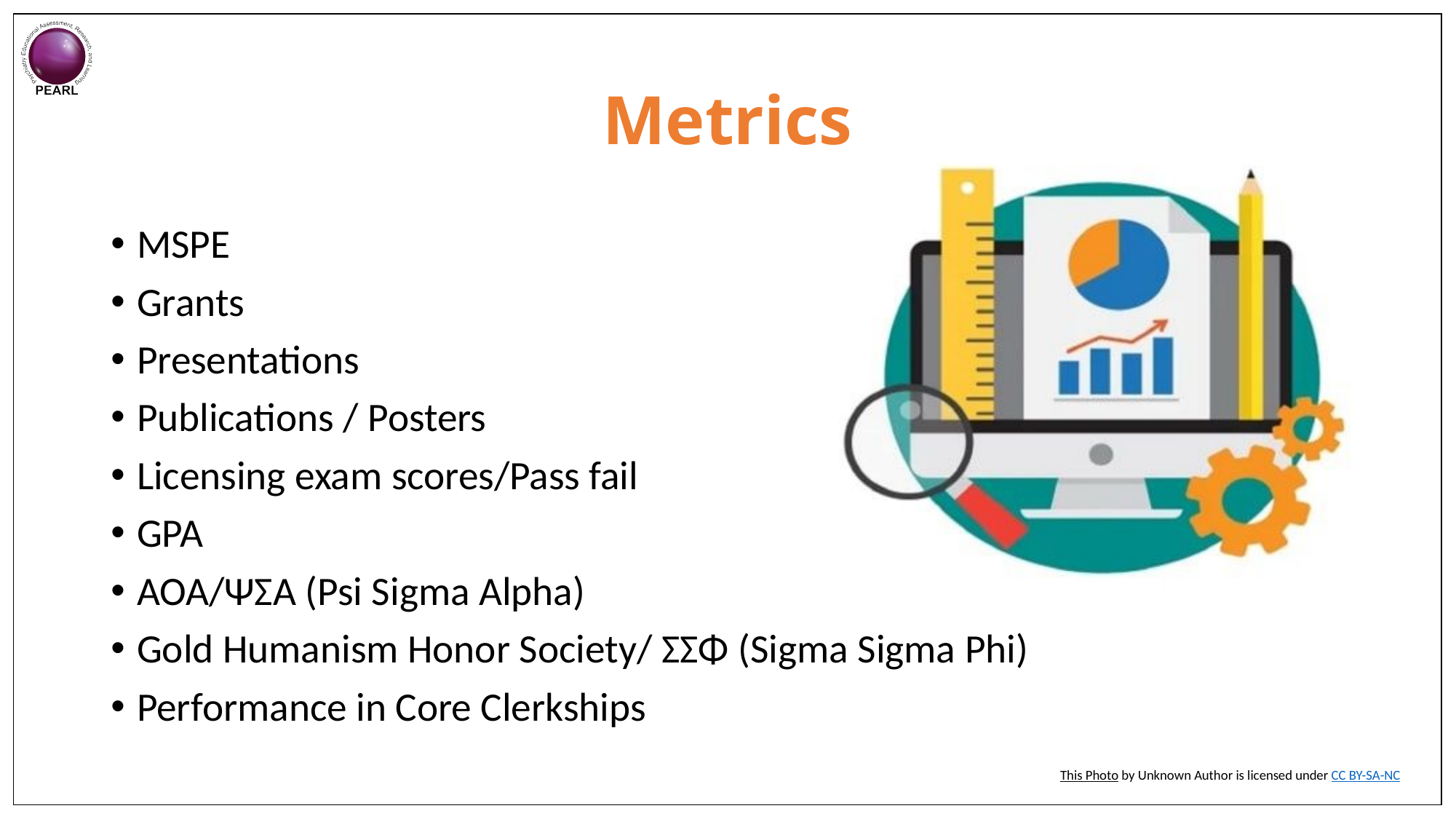

# Metrics
MSPE
Grants
Presentations
Publications / Posters
Licensing exam scores/Pass fail
GPA
AOA/ΨΣA (Psi Sigma Alpha)
Gold Humanism Honor Society/ ΣΣΦ (Sigma Sigma Phi)
Performance in Core Clerkships
This Photo by Unknown Author is licensed under CC BY-SA-NC

## Slide 17
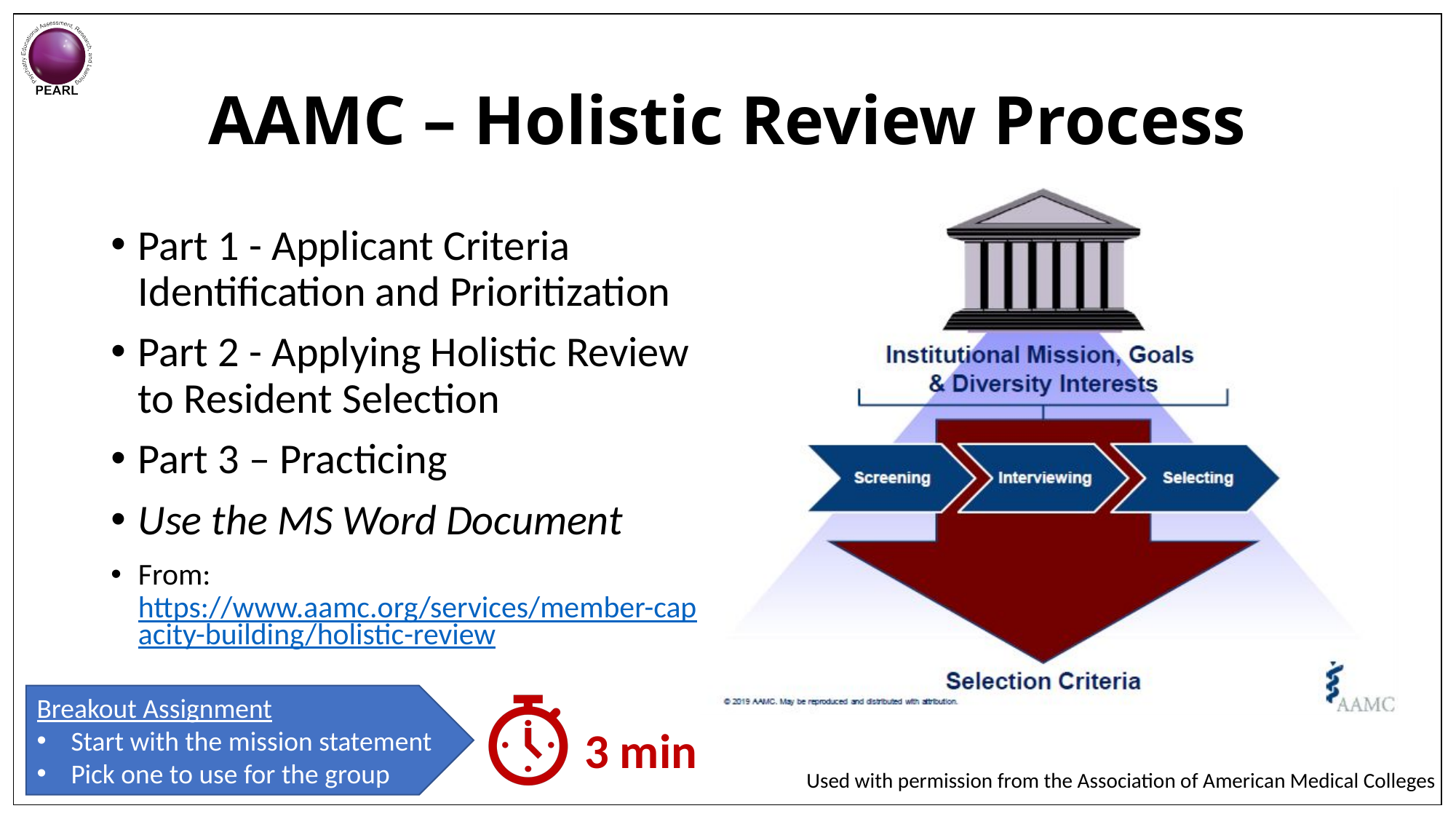

# AAMC – Holistic Review Process
Part 1 - Applicant Criteria Identification and Prioritization
Part 2 - Applying Holistic Review to Resident Selection
Part 3 – Practicing
Use the MS Word Document
From: https://www.aamc.org/services/member-capacity-building/holistic-review
Breakout Assignment
Start with the mission statement
Pick one to use for the group
3 min
Used with permission from the Association of American Medical Colleges

## Slide 18
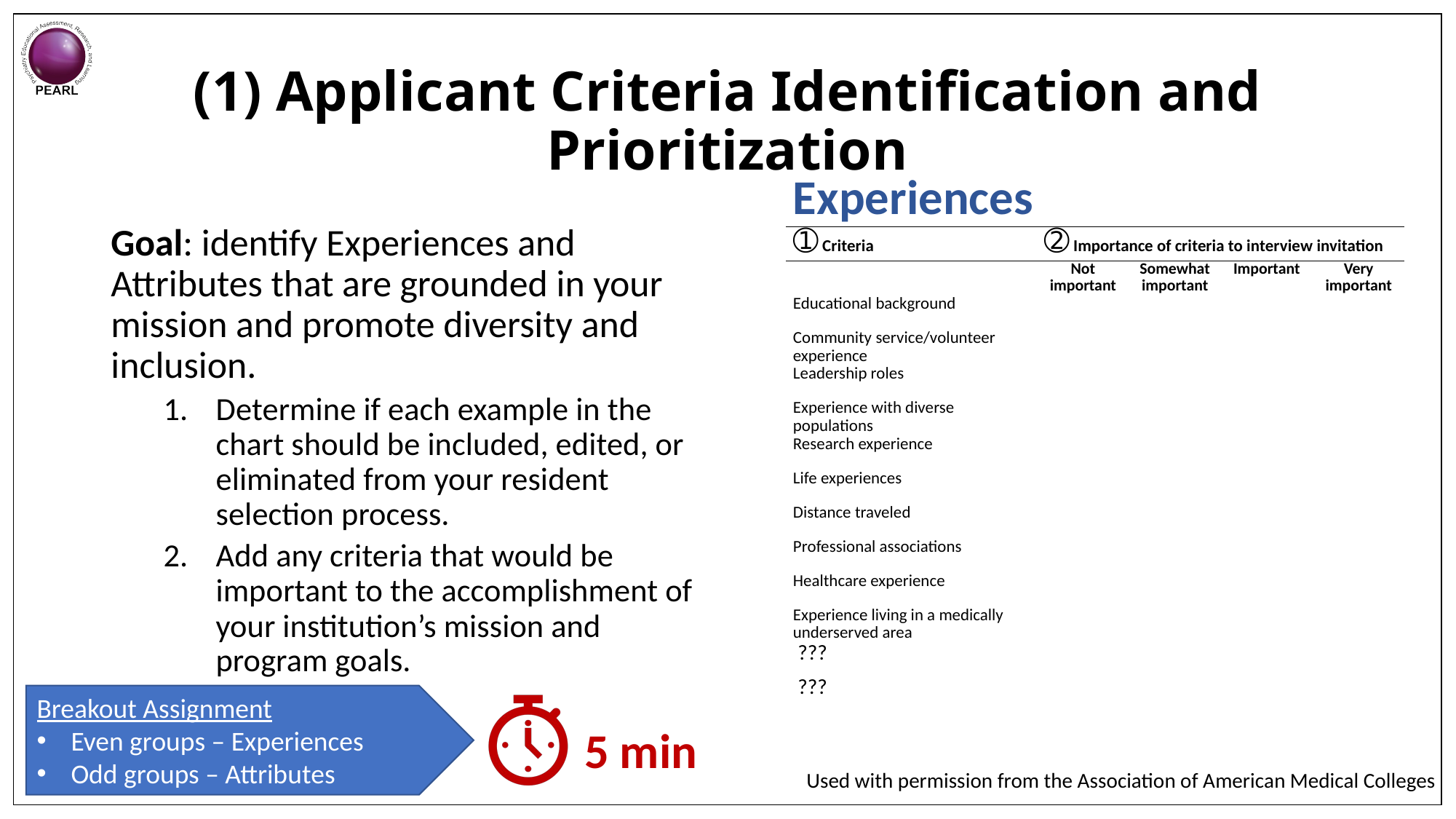

# (1) Applicant Criteria Identification and Prioritization
| Experiences | | | | |
| --- | --- | --- | --- | --- |
| ➀ Criteria | ➁ Importance of criteria to interview invitation | | | |
| | Not important | Somewhat important | Important | Very important |
| Educational background | | | | |
| Community service/volunteer experience | | | | |
| Leadership roles | | | | |
| Experience with diverse populations | | | | |
| Research experience | | | | |
| Life experiences | | | | |
| Distance traveled | | | | |
| Professional associations | | | | |
| Healthcare experience | | | | |
| Experience living in a medically underserved area | | | | |
| ??? | | | | |
| ??? | | | | |
Goal: identify Experiences and Attributes that are grounded in your mission and promote diversity and inclusion.
Determine if each example in the chart should be included, edited, or eliminated from your resident selection process.
Add any criteria that would be important to the accomplishment of your institution’s mission and program goals.
Breakout Assignment
Even groups – Experiences
Odd groups – Attributes
5 min
Used with permission from the Association of American Medical Colleges

## Slide 19
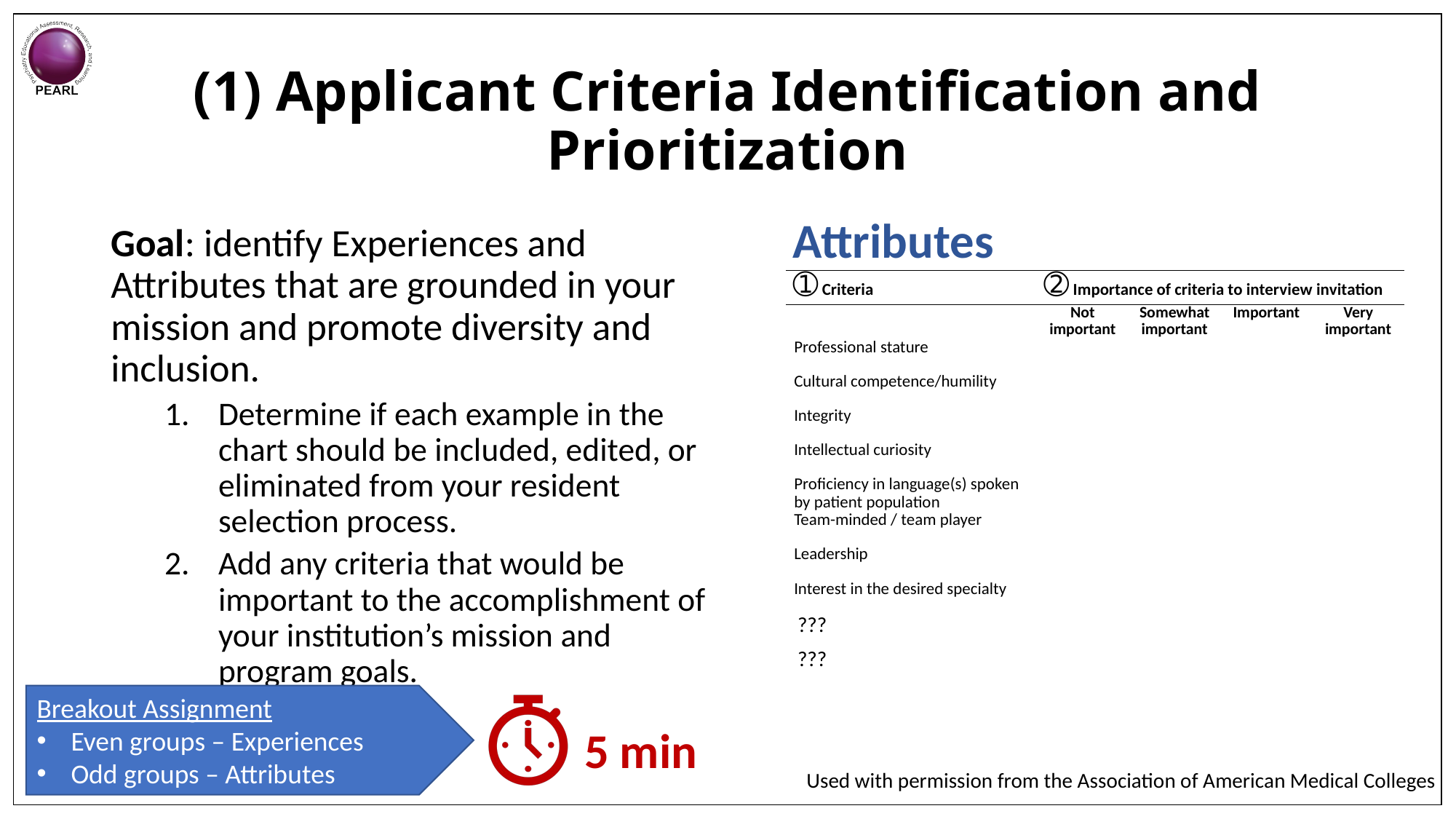

# (1) Applicant Criteria Identification and Prioritization
Goal: identify Experiences and Attributes that are grounded in your mission and promote diversity and inclusion.
Determine if each example in the chart should be included, edited, or eliminated from your resident selection process.
Add any criteria that would be important to the accomplishment of your institution’s mission and program goals.
| Attributes | | | | |
| --- | --- | --- | --- | --- |
| ➀ Criteria | ➁ Importance of criteria to interview invitation | | | |
| | Not important | Somewhat important | Important | Very important |
| Professional stature | | | | |
| Cultural competence/humility | | | | |
| Integrity | | | | |
| Intellectual curiosity | | | | |
| Proficiency in language(s) spoken by patient population | | | | |
| Team-minded / team player | | | | |
| Leadership | | | | |
| Interest in the desired specialty | | | | |
| ??? | | | | |
| ??? | | | | |
Breakout Assignment
Even groups – Experiences
Odd groups – Attributes
5 min
Used with permission from the Association of American Medical Colleges

## Slide 20
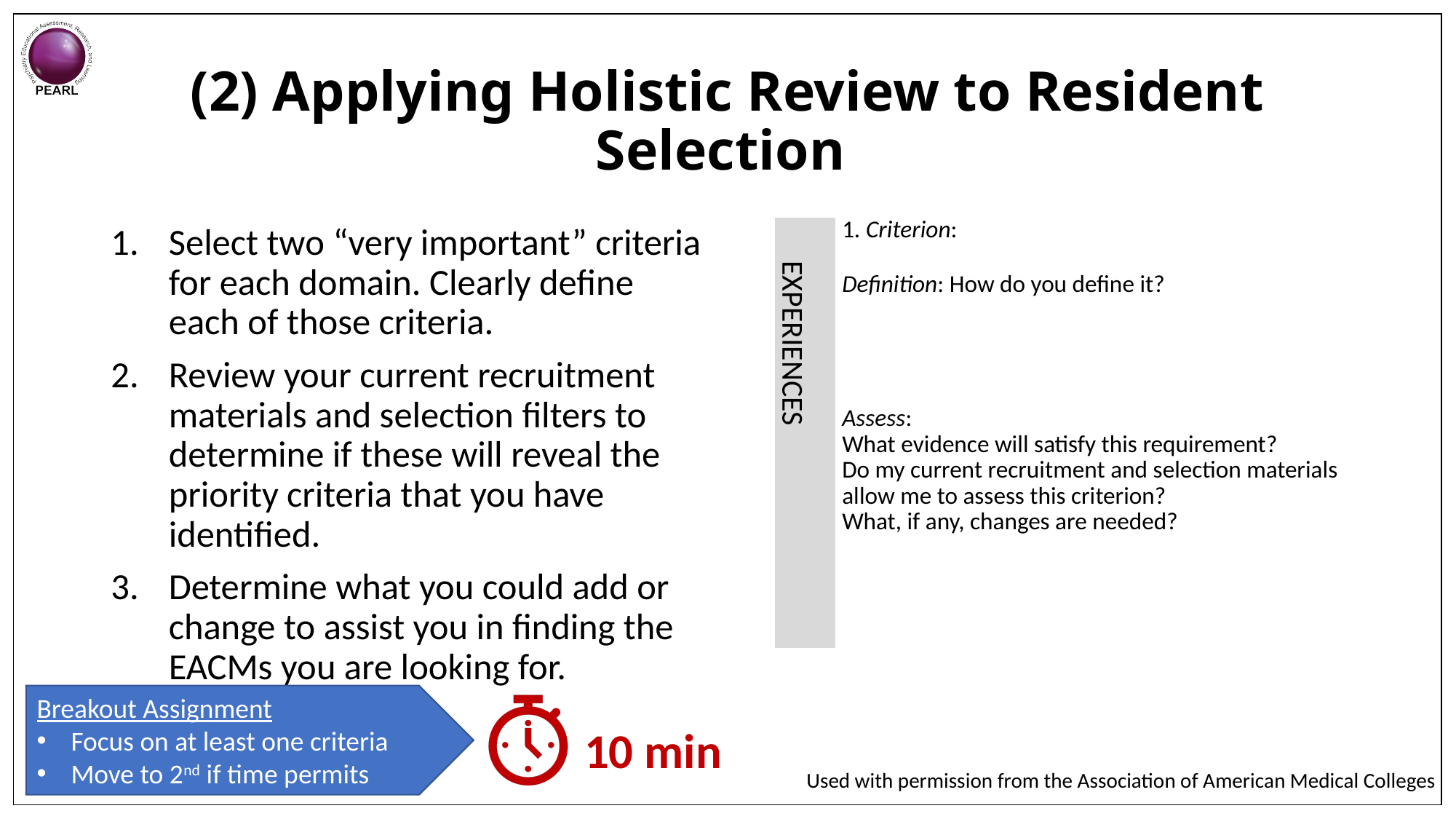

# (2) Applying Holistic Review to Resident Selection
| EXPERIENCES | 1. Criterion: |
| --- | --- |
| | Definition: How do you define it? |
| | Assess: What evidence will satisfy this requirement? Do my current recruitment and selection materials allow me to assess this criterion? What, if any, changes are needed? |
Select two “very important” criteria for each domain. Clearly define each of those criteria.
Review your current recruitment materials and selection filters to determine if these will reveal the priority criteria that you have identified.
Determine what you could add or change to assist you in finding the EACMs you are looking for.
Breakout Assignment
Focus on at least one criteria
Move to 2nd if time permits
10 min
Used with permission from the Association of American Medical Colleges

## Slide 21
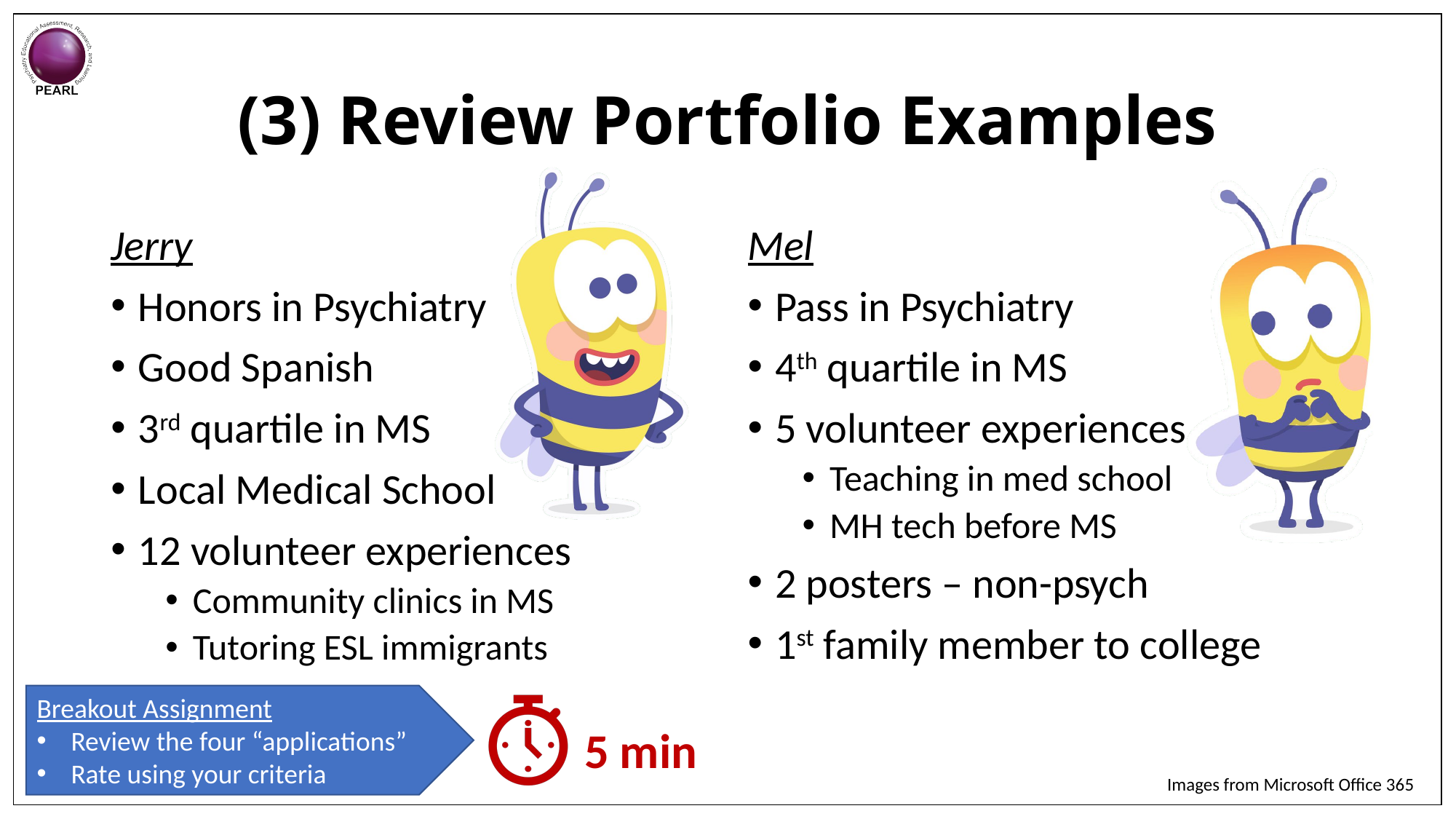

# (3) Review Portfolio Examples
Jerry
Honors in Psychiatry
Good Spanish
3rd quartile in MS
Local Medical School
12 volunteer experiences
Community clinics in MS
Tutoring ESL immigrants
Mel
Pass in Psychiatry
4th quartile in MS
5 volunteer experiences
Teaching in med school
MH tech before MS
2 posters – non-psych
1st family member to college
Breakout Assignment
Review the four “applications”
Rate using your criteria
5 min
Images from Microsoft Office 365

## Slide 22
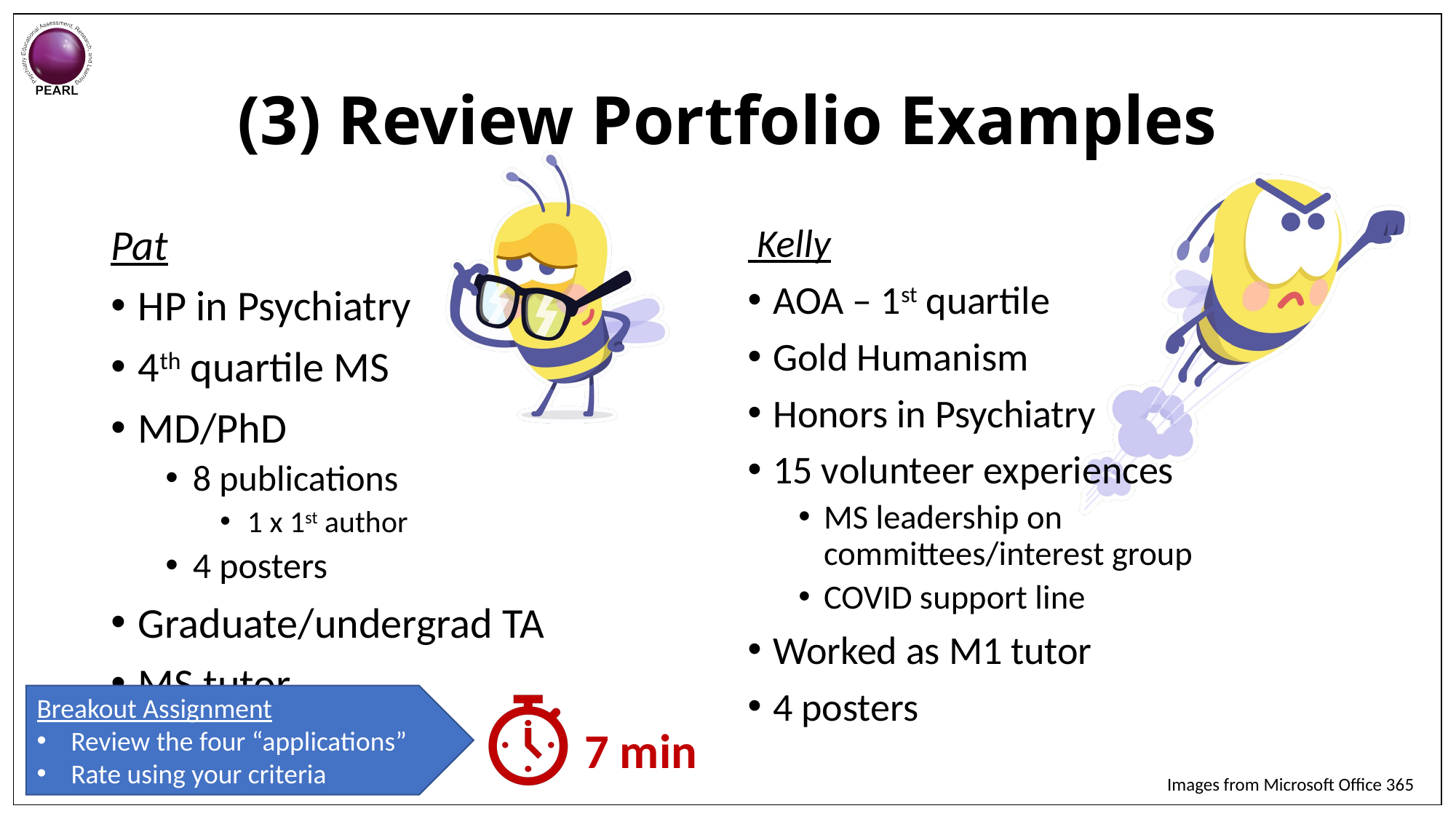

# (3) Review Portfolio Examples
Pat
HP in Psychiatry
4th quartile MS
MD/PhD
8 publications
1 x 1st author
4 posters
Graduate/undergrad TA
MS tutor
 Kelly
AOA – 1st quartile
Gold Humanism
Honors in Psychiatry
15 volunteer experiences
MS leadership on committees/interest group
COVID support line
Worked as M1 tutor
4 posters
Breakout Assignment
Review the four “applications”
Rate using your criteria
7 min
Images from Microsoft Office 365

## Slide 23
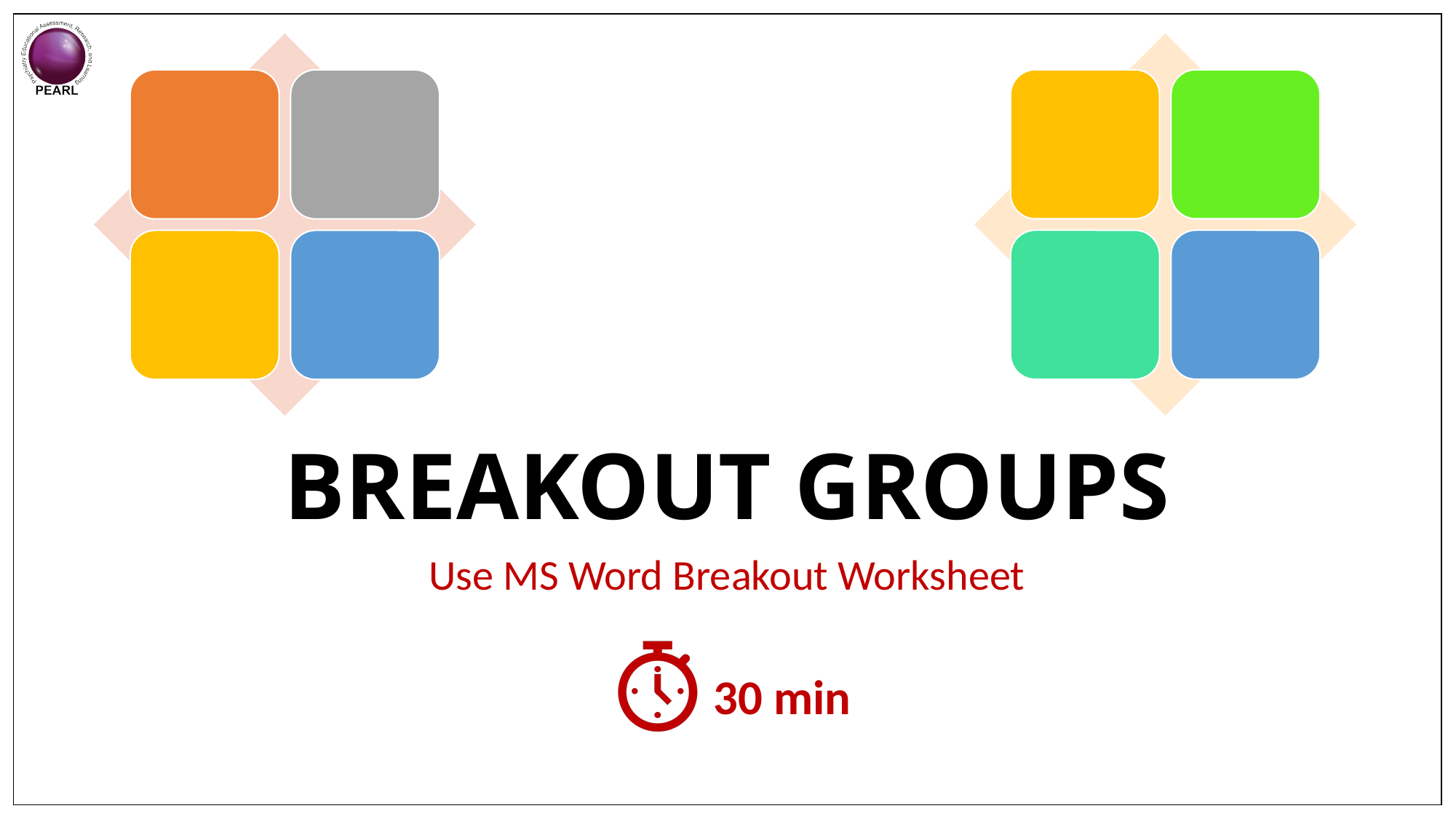

# BREAKOUT GROUPS
Use MS Word Breakout Worksheet
30 min

## Slide 24
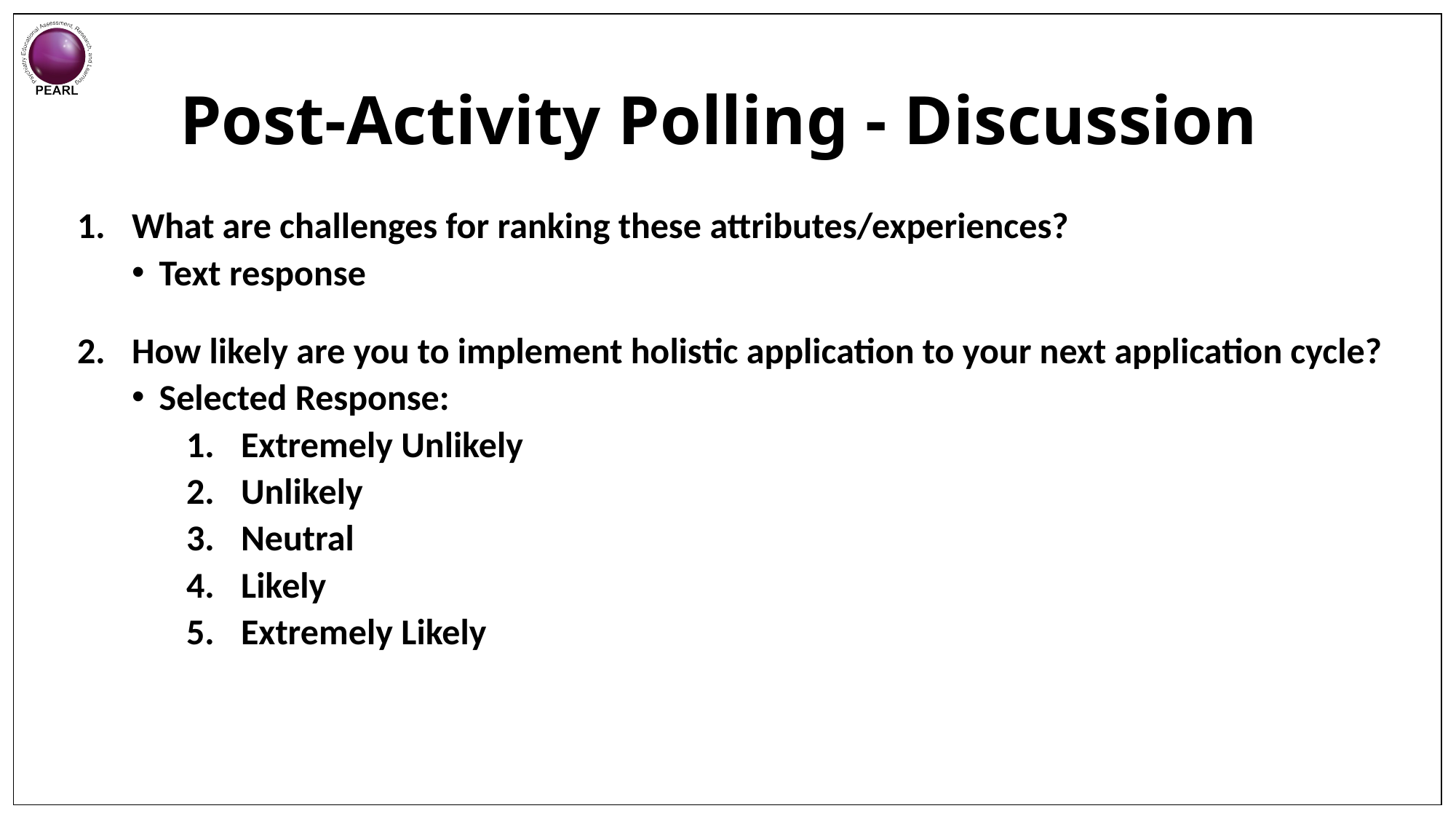

# Post-Activity Polling - Discussion
What are challenges for ranking these attributes/experiences?
Text response
How likely are you to implement holistic application to your next application cycle?
Selected Response:
Extremely Unlikely
Unlikely
Neutral
Likely
Extremely Likely

## Slide 25
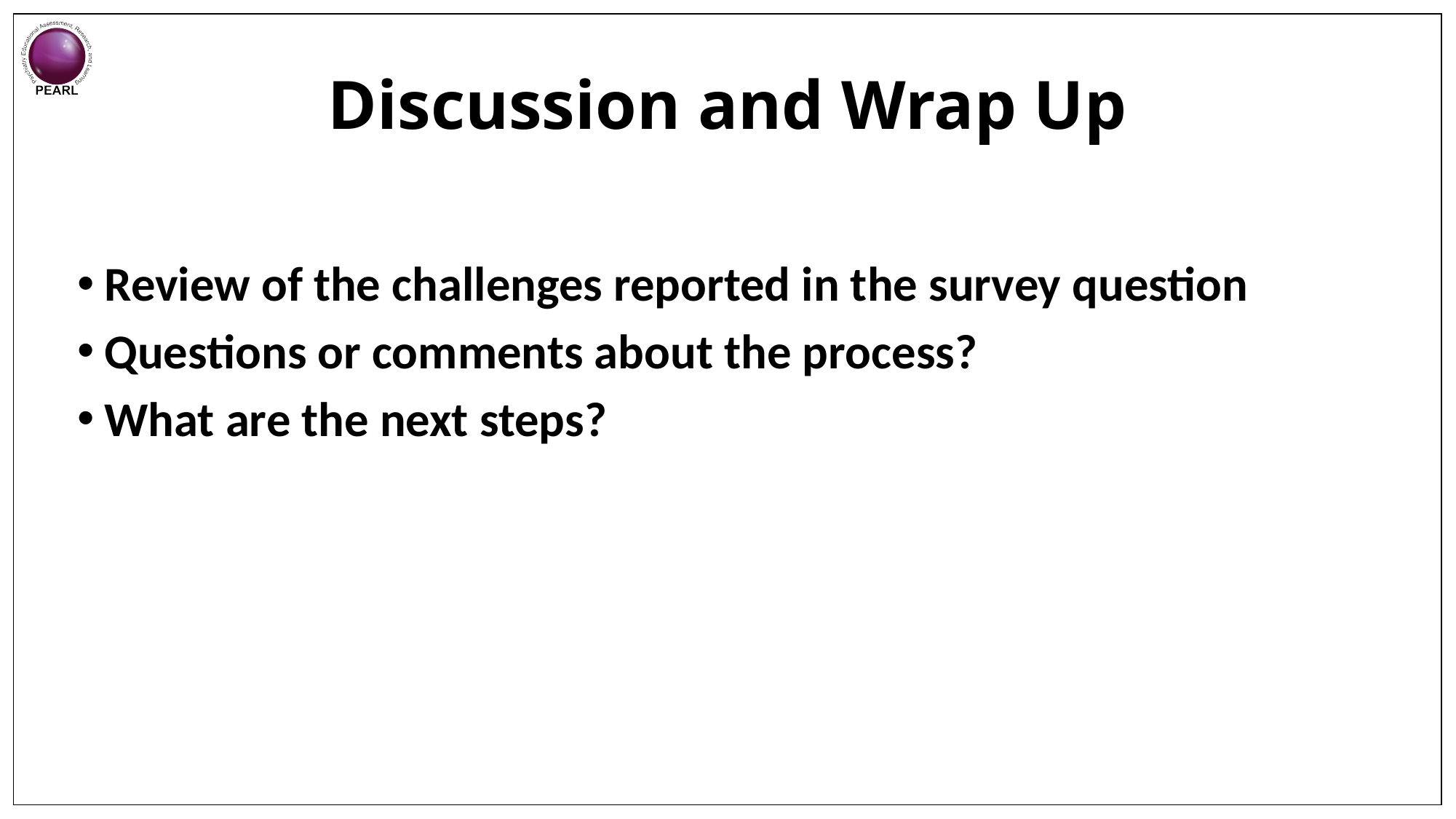

# Discussion and Wrap Up
Review of the challenges reported in the survey question
Questions or comments about the process?
What are the next steps?
