# Supplementary material for: Implementation of the AAMC's Holistic Review Model for Psychiatry Resident Recruitment
Source: MedEdPORTAL. 2023 Feb 7;19:11299. doi: 10.15766/mep_2374-8265.11299 (PMC9902530; doi:10.15766/mep_2374-8265.11299)
Supplement: Supplementary file 1 — Holistic Review Didactic Slides.pptxBreakout Group Exercise Worksheet.docxApplicant Criteria Identification and Prioritization.docxApplying Holistic Review to Resident Selection.docxSurvey.docx [file mep_2374-8265.11299-s001.zip › B. Breakout Group Exercise Worksheet.docx]

#
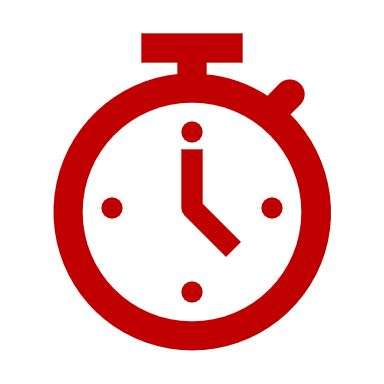
PEARL Holistic Selection – Breakout Activity

**3 min**

# Organize and Select a Mission Statement (Part 0)

# *Select a timekeeper for the group*

# *Select a reporter/scribe for the group*

# *Select a mission statement to use for this activity, either:*

# *Use your own and collaborate on assessments*

# *Use one for the entire group and collaborate on criteria and assessments*

#
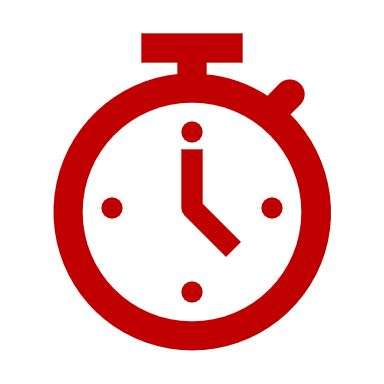


**5 min**

# Applicant Criteria Identification and Prioritization (Part 1)

**Purpose:** A critical part of a holistic selection process is identifying Experiences, Attributes, Competencies, and Metrics (EACMs) that are grounded in your mission and promote diversity and inclusion. Developing a shared understanding of how these criteria are prioritized facilitates recruitment, helps orient reviewers and interviewers, and informs the development of evaluation rubrics.

*The group should decide to work on either Experiences or Metrics. If you have time, you can return to the other. Make your choice!*

This activity will help you to “widen the lens” through which you assess residents by identifying and ranking the mission-driven EACMs that would add value to your program. For this workshop, we will limit to Experiences and Metrics.

**Directions:** For each applicant criterion:

**Part 1**

1. Determine if each example in the following charts should be included, edited, or eliminated from your resident selection process.
2. Add any criteria that would be important to the accomplishment of your institution’s mission and program goals.

**Part 2**

1. Rank how the EACMs contribute to your decision to invite a resident for an interview.

## Experiences

| ➀ **Criteria** | ➁ **Importance of criteria to interview invitation** | | | |
| --- | --- | --- | --- | --- |
|  | **Not important** | **Somewhat important** | **Important** | **Very important** |
| Educational background |  |  |  |  |
| Community service/volunteer experience |  |  |  |  |
| Leadership roles |  |  |  |  |
| Experience with diverse populations |  |  |  |  |
| Research experience |  |  |  |  |
| Life experiences |  |  |  |  |
| Distance traveled |  |  |  |  |
| Professional associations |  |  |  |  |
| Healthcare experience |  |  |  |  |
| Experience living in a medically underserved area |  |  |  |  |
|  |  |  |  |  |
|  |  |  |  |  |
|  |  |  |  |  |
|  |  |  |  |  |
|  |  |  |  |  |
|  |  |  |  |  |

**Attributes**

**Note:** If these metrics are not available to you, please edit, delete, and/or add any alternatives.

| ➀ **Criteria** | ➁ **Importance of criteria to interview invitation** | | | |
| --- | --- | --- | --- | --- |
|  | **Not important** | **Somewhat important** | **Important** | **Very important** |
| Professional stature |  |  |  |  |
| Cultural competence/humility |  |  |  |  |
| Integrity |  |  |  |  |
| Intellectual curiosity |  |  |  |  |
| Proficiency in language(s) spoken by patient population |  |  |  |  |
| Team-minded / team player |  |  |  |  |
| Leadership |  |  |  |  |
| Interest in the desired specialty |  |  |  |  |
| Professional stature |  |  |  |  |
| Cultural competence/humility |  |  |  |  |
| Integrity |  |  |  |  |
| Intellectual curiosity |  |  |  |  |
| Proficiency in language(s) spoken by patient population |  |  |  |  |
| Team-minded / team player |  |  |  |  |
| Leadership |  |  |  |  |
| Interest in the desired specialty |  |  |  |  |
| Professional stature |  |  |  |  |
|  |  |  |  |  |
|  |  |  |  |  |
|  |  |  |  |  |
|  |  |  |  |  |


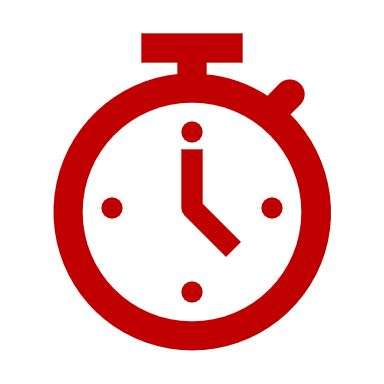


**10 min**

# Applying Holistic Review to Resident Selection (Part 2)

**Purpose:** Developing shared definitions of the criteria you identified in Activity 1 helps orient reviewers and interviewers and informs the development of evaluation rubrics; it can also help mitigate the influence of unconscious bias. This activity will help you to define your high-priority criteria and assess if your recruitment materials and selection processes reflect your priorities.

**Directions:**

1. Review your rankings from Activity 1 and select two “very important” criteria for each of the four domains in the EACM model. Clearly define each of those criteria.
2. Look at your current recruitment materials and selection filters to determine if these will reveal the priority criteria that you have identified.
3. Determine what you could add or change to assist you in finding the EACMs you are looking for.

## Part 1: Resident Selection Criteria

| **EXPERIENCES** | **1.** *Criterion:* |
| --- | --- |
|  | **Definition:** *How do you define it?* |
|  | **Assess:** *What evidence will satisfy this requirement? Do my current recruitment and selection materials allow me to assess this criterion? What, if any, changes are needed?* |
|  | **2.** *Criterion:* |
|  | **Definition:** *How do you define it?* |
|  | **Assess:** *What evidence will satisfy this requirement? Do my current recruitment and selection materials allow me to assess this criterion? What, if any, changes are needed?* |

**Notes:**

## Resident Selection Criteria (continued)

| **ACADEMIC METRICS** | **1.** *Criterion:* |
| --- | --- |
|  | **Definition:** *How do you define it?* |
|  | **Assess:** *What evidence will satisfy this requirement? Do my current recruitment and selection materials allow me to assess this criterion? What, if any, changes are needed?* |
|  | **2.** *Criterion:* |
|  | **Definition:** *How do you define it?* |
|  | **Assess:** *What evidence will satisfy this requirement? Do my current recruitment and selection materials allow me to assess this criterion? What, if any, changes are needed?* |

**Notes:**


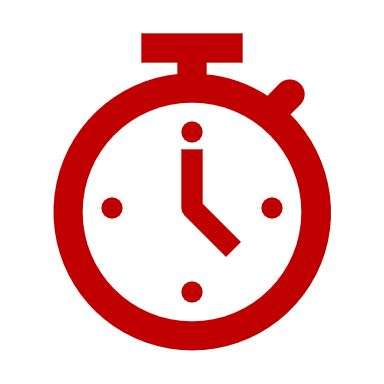
**Rating Portfolio Examples (Part 3)**

**7 min**

**Directions**:

1. Copy the criterion you determined in Activity 2 the spaces below
2. Review the Minon’s mini-application and determine your assessment of the criterion.

*Criterion 1*:

*Criterion 2*:

| *Pat*   - HP in Psychiatry - 4^th^ quartile MS – mid-tier - MD/PhD - immunology   - 8 publications – none in psychiatry     - 2 x 1^st^ author   - 4 posters – none in psychiatry - 5 volunteer experiences   - MS tutor   - Research mentor - Work experience   - Graduate/undergrad TA - Grew up in a small town, undergrad and MS in mid-sized urban center | *Assessent of Criterion 1*:  *Assessment of Criterion 2*: | 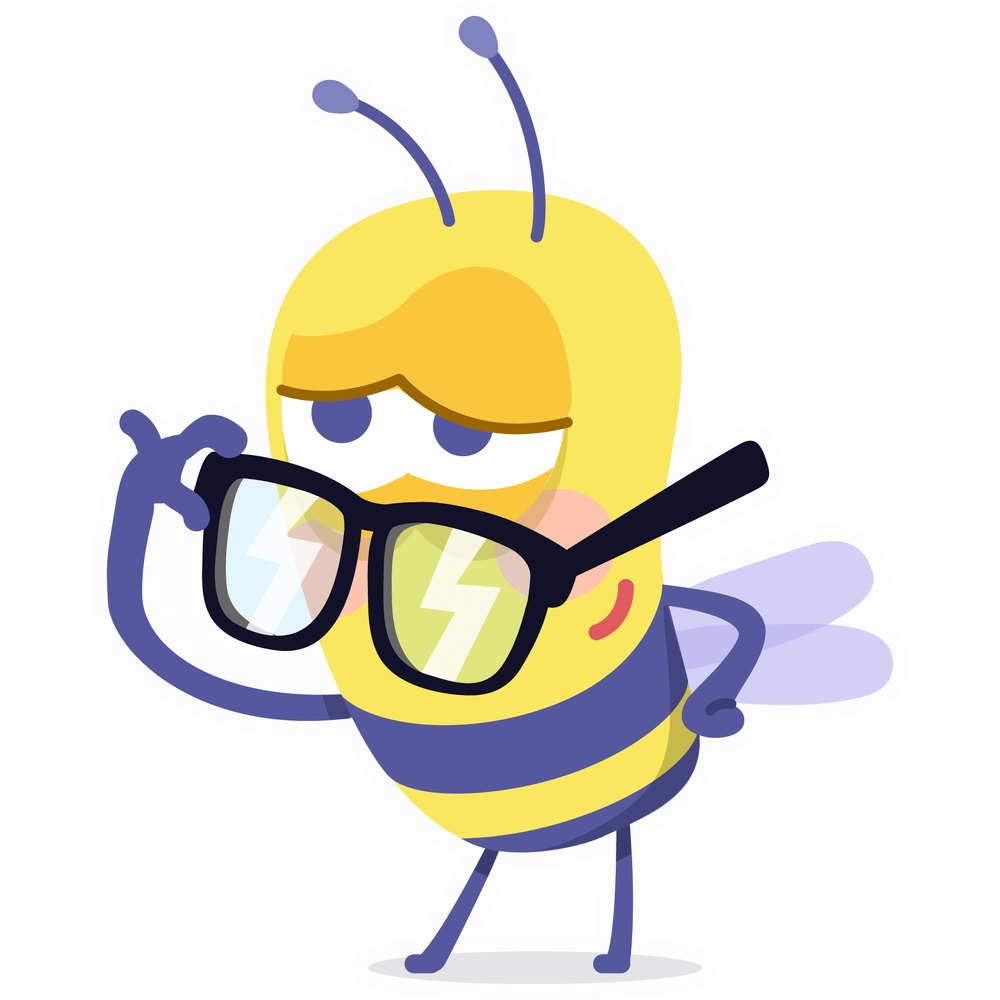 |
| --- | --- | --- |
|  |  |  |
| Kelly   - AOA – 1^st^ quartile in MS – upper-tier - Gold Humanism - Honors in Psychiatry - 15 volunteer experiences   - MS leadership on committees/interest group   - COVID support line - Work experience   - M1 tutor - 4 posters - Grew up in small rural town - Undergrad and MS in same town | *Assessent of Criterion 1*:  *Assessment of Criterion 2*: | 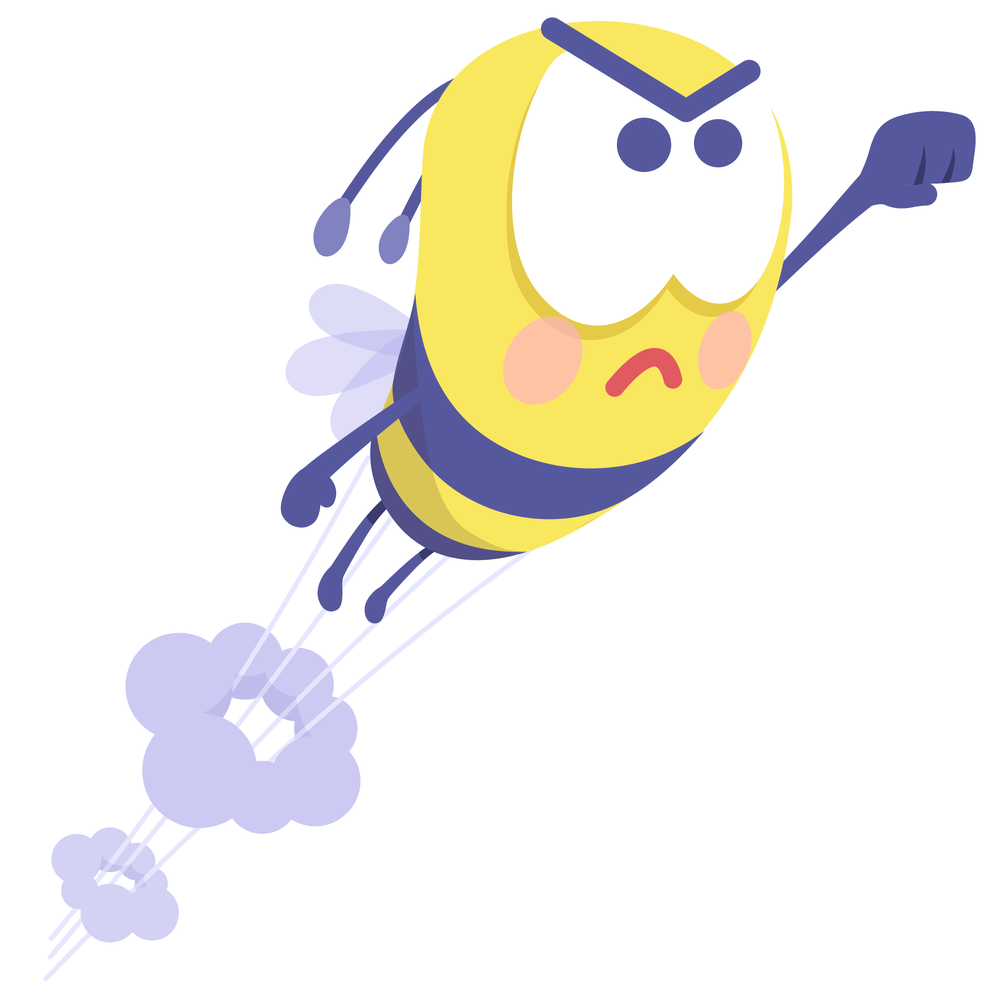 |
|  |  |  |
| *Jerry*   - Honors in Psychiatry - Good Spanish - 3^rd^ quartile in MS – local region – mid-tier - 12 volunteer experiences   - Community clinics in MS   - Tutoring ESL immigrants   - Mission abroad to Central America - Work experience   - Case worker for community psych agency   - CNA – nursing home - 5 posters – 2 x psych - Second generation immigrant | *Assessent of Criterion 1*:  *Assessment of Criterion 2*: | 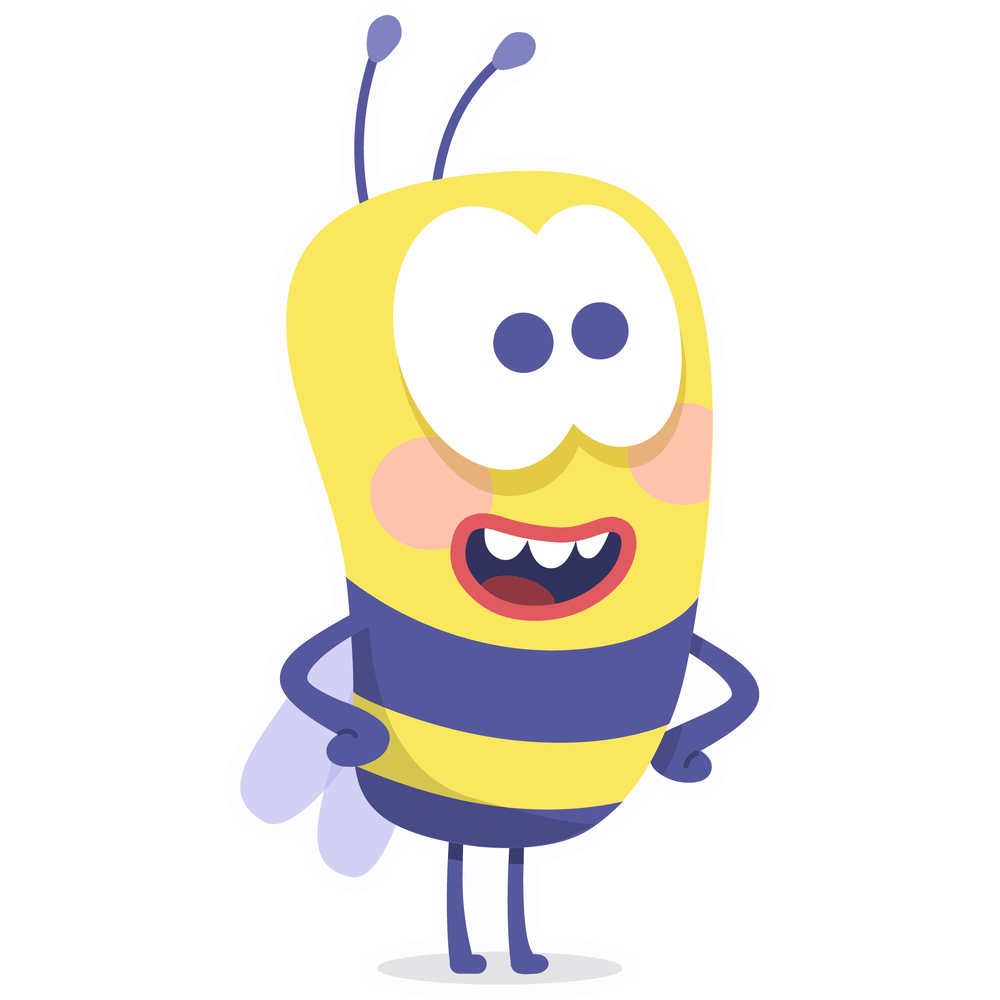 |
|  |  |  |
| *Mel*   - Pass in Psychiatry - Failed USMLE 1 x 1 - 4^th^ quartile in MS – upper-tier - 5 volunteer experiences   - Teaching in med school   - Community free clinic - Work experience   - Mental health tech - undergraduate - 2 posters – QI projects in psychiatry - 1^st^ family member to go to college - Grew up in large urban center - Undergrad and MS in different parts of the country - Undergrad degree in psychology | *Assessent of Criterion 1*:  *Assessment of Criterion 2*: | 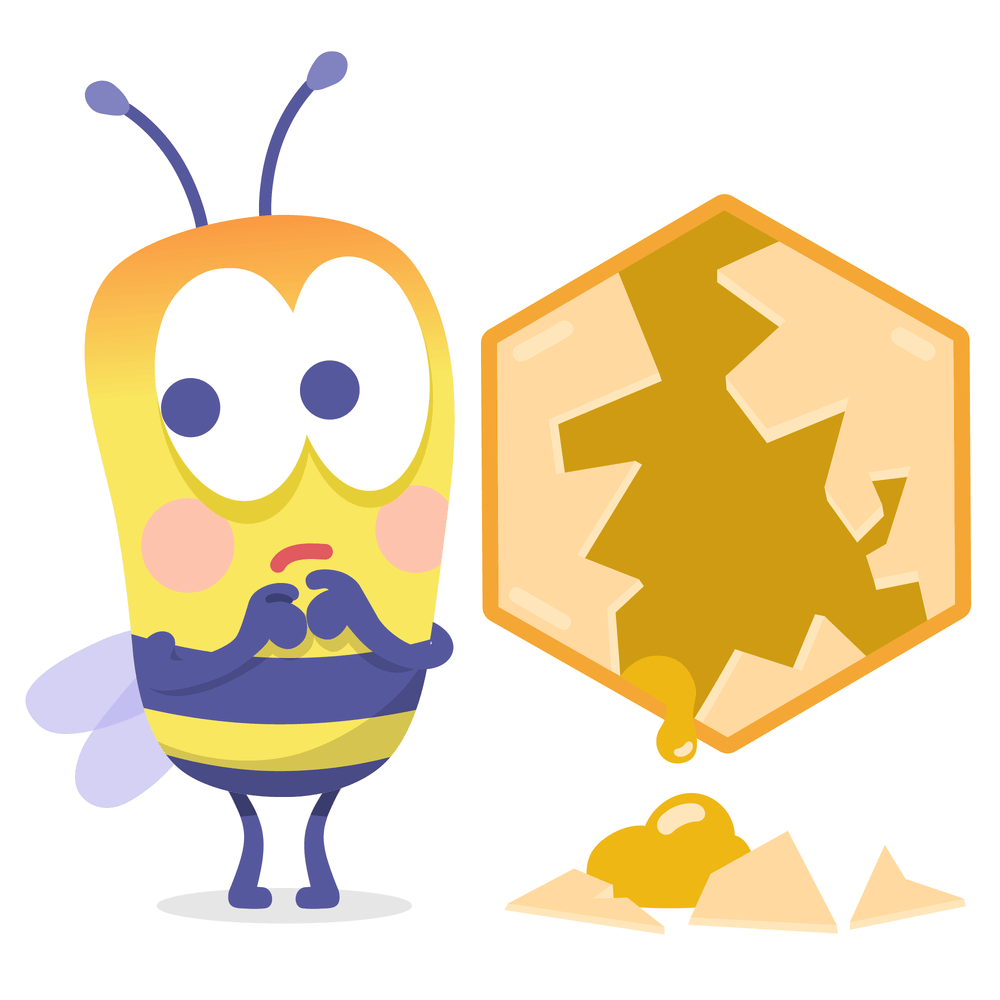 |
|  |  | Images from Microsoft Office 365 |


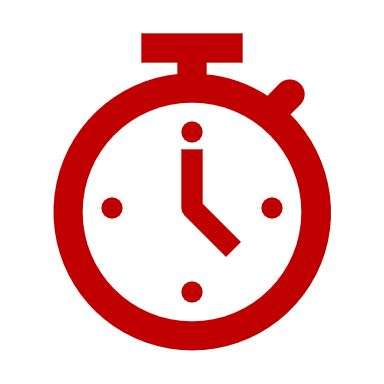
**Reflection (Part 4)**

**5 min**

Directions:

1. Reflect on how your group navigated the activity
2. As a group, summarize what challenges you encountered in ranking these attributes.
3. Select a spokesperson to present the findings to the larger group.

Challenges:
