# Supplementary material for: Implementation of the AAMC's Holistic Review Model for Psychiatry Resident Recruitment
Source: MedEdPORTAL. 2023 Feb 7;19:11299. doi: 10.15766/mep_2374-8265.11299 (PMC9902530; doi:10.15766/mep_2374-8265.11299)
Supplement: Supplementary file 1 — Holistic Review Didactic Slides.pptxBreakout Group Exercise Worksheet.docxApplicant Criteria Identification and Prioritization.docxApplying Holistic Review to Resident Selection.docxSurvey.docx [file mep_2374-8265.11299-s001.zip › Educational Summary Report_11299.pdf]

MedEdPORTAL® The AAMC Journal of Teaching and Learning Resources

Original Publication Open Access

Implementation of the AAMC's Holistic Review Model for Psychiatry Resident Recruitment

Robert Marvin, MD*, Yoon Soo Park, PhD, Michael Dekhtyar, MEd, Marla Hartzen, MD, Laurel Bessey, MD, Ryan Finkenbine, MD, Brett Lloyd, MD, PhD, Senada Bajmakovic-Kacila, MD, Art Walaszek, MD, Ara Tekian, PhD

*Corresponding author: rwmarvin@uic.edu

Abstract

Introduction: In psychiatry, several converging factors are impacting the recruitment of residents: the increased competitiveness of the specialty, the national trend to take active steps to improve diversity and inclusion, and the decision from USMLE to change Step 1 to a pass/fail result. **Methods:** We developed a workshop for psychiatry residency program directors to meet these challenges and transition into using a holistic review model during recruitment. The workshop included (1) a didactic session providing background on the AAMC holistic review model; (2) a small-group exercise to determine and prioritize experiences, attributes, competencies, and metrics (EACMs) aligned with the program's mission and aims; (3) a review of the rankings from the previous exercise, selection of two "very important" criteria for each of the four domains of the EACM model, and operationalization of these criteria based on the recruitment process; and (4) a discussion focused on application of program criteria with example applicants. **Results:** The holistic review workshop was conducted at the American Association of Psychiatry Residency Directors conference in 2021 with 48 self-selected attendees. Following the workshop, 74% of attendees reported a likelihood of implementing holistic applications during their next application cycle, 78% were able to leave with at least one actionable item, 100% thought that the session was interactive, and 78% felt that the session met their expectations. **Discussion:** Implementing a holistic review for psychiatry residency recruitment can assist programs in responding to the rapidly changing landscape and achieve aims for improving diversity and inclusion.

Keywords
Holistic Review, Admissions/Selection, Psychiatry, Recruitment, Residency Preparation, Diversity, Equity, Inclusion

Educational Objectives

By the end of this activity, learners will be able to:

1. Define and operationalize holistic resident selection.
2. Use program-specific mission statement and aims to identify and balance selection metrics.
3. Develop and implement screening rating instruments.

Introduction

Resident selection practices in psychiatry have long relied on using licensure examination scores as the basis for identifying the initial cohort for interviews. The 2021 national survey by the National Resident Matching Program has shown that United States Medical Licensing Examination (USMLE) scores remain the focus of initial selection,[1] reinforcing a similar survey finding among psychiatry training directors.[2] This focus on licensure scores has been associated with neglect for holistic characteristics such as an applicant's experiences, attributes, and academic achievements. Importantly, narrow reliance on test scores may demonstrate indifference toward valued aspects of diversity and inclusion in psychiatry training and the practicing workforce.[3] The field of psychiatry is facing new challenges as it embraces a larger, more competitive applicant pool while considering nontraditional competencies for training the next generation of psychiatrists.[4] Studies have also shown that predictive qualities of the USMLE are mixed, with poor association between USMLE scores and clinical performance and professionalism.[5] Starting in January 2022, the National Board of Medical Examiners and the Federation of State Medical Board began reporting the Step 1 score as pass or fail only.[6] Additionally, preliminary data suggest that a holistic review process can increase diversity in the applicant pool.[7]

Citation:
Marvin R, Park YS, Dekhtyar M, et al. Implementation of the AAMC's holistic review model for psychiatry resident recruitment. *MedEdPORTAL*. 2023;19:11299.
https://doi.org/10.15766/mep_2374-8265.11299

Copyright © 2023 Marvin et al. This is an open-access publication distributed under the terms of the Creative Commons Attribution license.

Prior publications have described programs that guide medical students through the residency match process.[8,9] However, tools that provide residency program directors with guidance in choosing applicants do not exist. The goal of our interactive workshop is to operationalize the AAMC holistic review model[10] so it can be implemented at each participant's training program. The holistic review model categorizes applicant characteristics into experiences, attributes, competencies, and metrics (EACMs) to serve as the foundation for operationalizing the selection process. Additionally, the workshop materials include references for expanding the holistic review during the interview process by using structured interviews/multiple mini-interviews and situational judgment testing tp supplement traditional metrics for selection.[11,12]

This workshop provides guidance and hopefully reduces anxiety for training directors and programs aiming to conduct a holistic review, delivers consensus-building methods and processes to identify institutional values, and facilitates translation of conceptual ideas to operationalized selections.

Methods

This workshop was developed as part of the educational mission of the Psychiatry Educational Assessment Research and Learning consortium[13] and was based on the experiences of several member programs during their implementation of a holistic review process. These programs developed their process independently while other programs had not yet started. This confirmed a knowledge gap within the consortium's member programs that was extrapolated to the psychiatry training programs at large. The workshop content was developed over a series of working meetings with the consortium members.

The workshop was conducted at the American Association of Directors of Psychiatric Residency Training (AADPRT) annual meeting held virtually in March 2021 and was limited to 1 hour in duration. The facilitators were all current or past psychiatry resident training directors.

The workshop was divided into four parts, each facilitated by one of the authors. Initially, using a PowerPoint slide deck (Appendix A), we provided a short didactic session (15 minutes) that covered the typical recruitment process in psychiatry, polling for current level of knowledge, and the AAMC holistic review model. We utilized the virtual conference platform to form breakout groups that included facilitators (30 minutes total). We tasked the groups with determining and prioritizing the experiences and attributes most aligned with the program's mission and aims to promote diversity and inclusion, using the breakout worksheet (Appendix B). The breakout groups were given Appendices C and D as reference materials. We requested that the groups review the rankings from the previous exercise and select two "very important" criteria from experiences and attributes. Participants were asked to operationalize these criteria based on their recruitment process, using Appendix B. The groups then practiced applying their criteria to example applicants, again using Appendix B. Finally, we reconvened the large group, led a debriefing discussion of the experience and process, and conducted final polling questions (15 minutes). Depending on the stage of implementation of the holistic review process, the debriefing could range from identifying areas of the recruitment process that needed revising to finalizing the criteria and operational methods. We posed several questions about the process, solicited challenges encountered, and explored the impact of bias. Additionally, we asked the group to consider the next steps in implementing the process.

As part of our implementation at the AADPRT annual meeting, we evaluated the participants during and after the workshop. We asked participants about their current status of using a holistic review process (measured at the beginning of the workshop) and their likelihood of implementing the holistic review process as a result of attending the workshop (measured toward the end of the workshop). A 5-point rating was used to collect the data using virtual survey tools. A postworkshop survey (Appendix E) on the effectiveness of the session was also administered by the conference administrators. We summarized participant responses using proportions. Differences in proportions were compared using the chi-square test. Data analyses were conducted using Stata 16 (StataCorp).

Results

Participants in this session included 48 psychiatric residency program directors who attended the virtual AADPRT meeting in March 2021 and self-selected to attend the workshop.

When polled about challenges to residency selection at their program related to the holistic review process, several respondents mentioned the large number of applications being reviewed. Additionally, attendees struggled with how to weight and prioritize different criteria, as well as how to account for interrater differences during the review process.

When the 48 participants were asked about their status of implementing a holistic review process, a majority did not know what holistic review was (31%), and equal amounts of participants had heard of it but had yet not started or had been using holistic review for over a year (22%). Lastly, some applicants had used

Copyright © 2023 Marvin et al. This is an open-access publication distributed under the terms of the Creative Commons Attribution license.

holistic review but had only begun implementing the process in the past year (25%). At the conclusion, a majority of attendees were either extremely likely (42%) or likely (32%) to implement holistic review during their next application cycle. Overall, the workshop significantly improved participant likelihood of implementing holistic review (increase of 21%, $p = .02$).

Regarding the overall quality of the session and activities as evaluated on the conference survey, a majority of attendees were able to leave with at least one actionable item (78%), thought that the session was interactive (100%), and felt that the session met their expectations (78%). Participants also commented on the practical utility of the workshop to enhance their experience and the ability to operationalize the concepts in practice. There were multiple comments on the practicality of the workshop, which allowed translation and implementation of holistic review into a program's selection process.

Discussion

With a rapidly evolving recruitment landscape in psychiatry, programs are looking to alternative models for screening and reviewing applications. These alterations in resident recruitment processes have been accelerated with the change in Step 1 scores to pass/fail status, as well as the recent discontinuation of the Step 2 Clinical Skills examinations, prompting programs to transform resident selection. The AAMC holistic review model provides a framework that aligns with a program's mission statement, reduces reliance on USMLE scores, and assists in managing the increasing numbers of well-qualified applicants to the specialty. Our workshop contributes to providing practical guidelines and operationalizing the holistic review process—using the AAMC holistic review model—translated for psychiatry residency programs. The process can also be implemented broadly for other specialties.

The workshop provides background information on holistic review, offers practical recommendations for structuring the review process, and gives participants an opportunity to apply their own criteria with example students through group discussions. Our evaluation indicated a significant increase in participants' likelihood of implementing a holistic review.

This workshop had several limitations. It was conducted virtually at a national meeting, which limited the total time available for small-group exercises and discussion. We were only able to work on experiences and attributes, leaving competencies and metrics for a future session. The virtual format limited the ability to comprehensively survey the participants, including obtaining demographic information for the participants and response rates for the conference questions. We also had a group of program directors familiar with the process leading the discussions and assisting in the breakout groups. Smaller programs implementing this workshop may need to seek additional support from their GME office or other specialty training programs. Despite these limitations, the virtual platform also showed promise for conducting this type of workshop and holistic review discussions, as evidenced by the evaluation components of the workshop.

Ideally, this framework would be implemented as a series of workshops with a recruitment committee consisting of program directors, faculty, residents, and program administrators. Programs typically hold a series of weekly meetings, each with specific goals: identification of the EACMs aligned with their mission and how to operationalize them, development of a holistic rubric to be used to screen applications, and rubric validation with screeners. Additional workshops focusing on rubric development and validation are needed.

The tangible benefits from this model can be directly measured by tracking metrics pre- and postimplementation. By following the model through each stage of the recruitment process from initial application to review, invitation, interviewing, ranking, and matching, opportunities are created for targeted interventions to further guide and enhance the holistic review process. This workshop provides value to programs looking for a mission-driven approach to applicant selection. Future plans for the workshop include porting to a multispecialty setting for use at the GME level.

In conclusion, despite requiring more preparation and organizational time, a holistic review model can be successfully implemented in a manner that fits the specific mission, needs, and resources of a program. Representatives from many different institutions have shown an interest and willingness to transition to holistic review practices. Additional workshops and evaluations are needed to provide further guidance and improvements.

Appendices

A. Holistic Review Didactic Slides.pptx

B. Breakout Group Exercise Worksheet.docx

C. Applicant Criteria Identification and Prioritization.docx

D. Applying Holistic Review to Resident Selection.docx

E. Survey.docx

All appendices are peer reviewed as integral parts of the Original Publication.

Copyright © 2023 Marvin et al. This is an open-access publication distributed under the terms of the Creative Commons Attribution license.

Robert Marvin, MD: Professor and Residency Training Director, Department of Psychiatry, University of Illinois College of Medicine at Chicago; ORCID: https://orcid.org/0000-0002-6186-8423

Yoon Soo Park, PhD: Associate Professor and Associate Head, Department of Medical Education, University of Illinois College of Medicine at Chicago; ORCID: https://orcid.org/0000-0001-8583-4335

Michael Dekhtyar, MEd: Research Associate, Department of Medical Education, University of Illinois College of Medicine at Chicago; ORCID: https://orcid.org/0000-0002-8548-3624

Marla Hartzen, MD: Psychiatry Residency Training Director and Medical Director, AMG Park Ridge Behavioral Health, Advocate Lutheran General Hospital

Laurel Bessey, MD: Assistant Professor and Associate Residency Training Director, Department of Psychiatry, University of Wisconsin School of Medicine and Public Health

Ryan Finkenbine, MD: Professor, Residency Training Director, and Chair, Department of Psychiatry, University of Illinois College of Medicine at Peoria

Brett Lloyd, MD, PhD: Assistant Professor and Residency Training Director, Department of Psychiatry & Behavioral Sciences, Northwestern University Feinberg School of Medicine; ORCID: https://orcid.org/0000-0002-8674-9842

Senada Bajmakovic-Kacila, MD: Residency Training Director and Medical Director, Adult Outpatient Psychiatry Clinic, Department of Psychiatry, RUSH University Medical Center

Art Walaszek, MD: Professor and Vice Chair for Education and Faculty Development, Department of Psychiatry, University of Wisconsin School of Medicine and Public Health; ORCID: https://orcid.org/0000-0001-9416-6105

Ara Tekian, PhD: Professor, Director of International Programs, and Associate Dean for the Office of International Education, Department of Medical Education, University of Illinois College of Medicine at Chicago; ORCID: https://orcid.org/0000-0002-9252-1588

Disclosures

None to report.

Funding/Support

This study was supported by a research award from the American Board of Psychiatry and Neurology to Dr. Robert Marvin.

Ethical Approval

The University of Illinois at Chicago Institutional Review Board deemed further review of this project not necessary.

References

1. National Resident Matching Program, Data Release and Research Committee. *Results of the 2021 NRMP Program Director Survey*. National Resident Matching Program; 2021. Accessed December 12, 2022. https://www.nrmp.org/wp-content/uploads/2021/11/2021-PD-Survey-Report-for-WWW.pdf
2. Chang AK, Morreale M, Balon R. Factors influencing psychiatry residency applicant selection for interview. *Acad Psychiatry*. 2017;41(3):438-439. https://doi.org/10.1007/s40596-017-0693-5
3. Conrad SS, Addams AN, Young GH. Holistic review in medical school admissions and selection: a strategic, mission-driven response to shifting societal needs. *Acad Med*. 2016;91(11): 1472-1474. https://doi.org/10.1097/ACM.0000000000001403
4. Walaszek A. Keep calm and recruit on: residency recruitment in an era of increased anxiety about the future of psychiatry. *Acad Psychiatry*. 2017;41(2):213-220. https://doi.org/10.1007/s40596-017-0664-x
5. Stephenson-Famy A, Houmard BS, Oberoi S, Manyak A, Chiang S, Kim S. Use of the interview in resident candidate selection: a review of the literature. *J Grad Med Educ*. 2015;7(4):539-548. https://doi.org/10.4300/JGME-D-14-00236.1
6. USMLE Step 1 transition to pass/fail only score reporting. United States Medical Licensing Examination. September 15, 2021. Accessed December 12, 2022. https://www.usmle.org/usmle-step-1-transition-passfail-only-score-reporting
7. Aibana O, Swails JL, Flores RJ, Love L. Bridging the gap: holistic review to increase diversity in graduate medical education. *Acad Med*. 2019;94(8):1137-1141. https://doi.org/10.1097/ACM.0000000000002779
8. Kost A, Huntington J, Cawse-Lucas J. Assessing and impressing: a guide for medical school seniors who are applying for a family medicine residency. *MedEdPORTAL*. 2013;9:9416. https://doi.org/10.15766/mep_2374-8265.9416
9. Donaldson K, Sakamuri S, Moore J, Everett EN. A residency interview training program to improve medical student confidence in the residency interview. *MedEdPORTAL*. 2020; 16:10917. https://doi.org/10.15766/mep_2374-8265.10917
10. Holistic review. Association of American Medical Colleges. Accessed December 12, 2022. https://www.aamc.org/services/member-capacity-building/holistic-review
11. Andolsek KM. Improving the Medical Student Performance Evaluation to facilitate resident selection. *Acad Med*. 2016; 91(11):1475-1479. https://doi.org/10.1097/ACM.0000000000001386
12. Patterson F, Roberts C, Hanson MD, et al. 2018 Ottawa consensus statement: selection and recruitment to the healthcare professions. *Med Teach*. 2018;40(11):1091-1101. https://doi.org/10.1080/0142159X.2018.1498589
13. PEARL Psychiatry Educational Assessment Research and Learning. Accessed December 12, 2022. http://psychpearl.org

Received: May 13, 2022
Accepted: November 1, 2022
Published: February 7, 2023

Copyright © 2023 Marvin et al. This is an open-access publication distributed under the terms of the Creative Commons Attribution license.
